# Conversion of Polyethylene Waste into Gaseous Hydrocarbons via Integrated Tandem Chemical–Photo/Electrocatalytic Processes

**DOI:** 10.1021/acscatal.1c02133

**Published:** 2021-07-09

**Authors:** Christian M. Pichler, Subhajit Bhattacharjee, Motiar Rahaman, Taylor Uekert, Erwin Reisner

**Affiliations:** Yusuf Hamied Department of Chemistry, University of Cambridge, Lensfield Road, Cambridge CB2 1EW, U.K.

**Keywords:** polyethylene, oxidative depolymerization, decarboxylation, photocatalysis, electrocatalysis

## Abstract

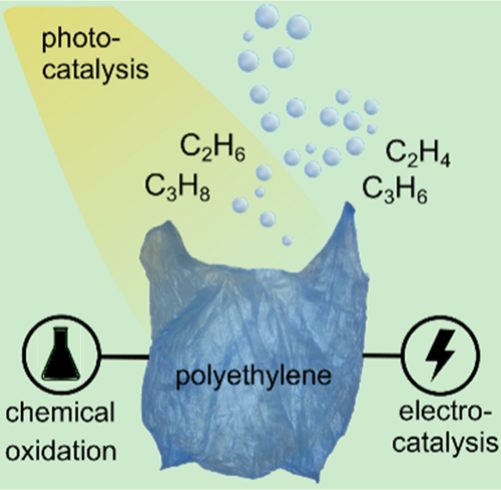

The chemical inertness of polyethylene makes chemical recycling challenging and motivates the development of new catalytic innovations to mitigate polymer waste. Current chemical recycling methods yield a complex mixture of liquid products, which is challenging to utilize in subsequent processes. Here, we present an oxidative depolymerization step utilizing diluted nitric acid to convert polyethylene into organic acids (40% organic acid yield), which can be coupled to a photo- or electrocatalytic decarboxylation reaction to produce hydrocarbons (individual hydrocarbon yields of 3 and 20%, respectively) with H_2_ and CO_2_ as gaseous byproducts. The integrated tandem process allows for the direct conversion of polyethylene into gaseous hydrocarbon products with an overall hydrocarbon yield of 1.0% for the oxidative/photocatalytic route and 7.6% for the oxidative/electrolytic route. The product selectivity is tunable with photocatalysis using TiO_2_ or carbon nitride, yielding alkanes (ethane and propane), whereas electrocatalysis on carbon electrodes produces alkenes (ethylene and propylene). This two-step recycling process of plastics can use sunlight or renewable electricity to convert polyethylene into valuable, easily separable, gaseous platform chemicals.

## Introduction

Reducing the amount of waste plastics and mitigating its disposal in landfills and escape into the environment are important contemporary challenges.^1^ Polyolefins such as polyethylene (PE) and polypropylene (PP) make up more than 50% of overall plastic production (380 Mt in 2015),^2^ but are particularly challenging to recycle economically due to their tendency to decrease in material quality with each mechanical recycling step.^3,4^ Current chemical waste processing for PE includes pyrolysis and gasification, which operate at high temperatures (500–1000 °C), yield a complex mixture of products that require subsequent upgrading, and typically rely on large-scale chemical plants or refineries.^5,6^

PE has been recently converted into shorter chain hydrocarbons by hydrogenolysis.^7^ It could also be converted to hydrogen and carbon nanotubes utilizing microwave heating.^8^ Other chemical recycling approaches oxidatively convert polymers into a range of products such as carboxylic acids.^9−14^ For example, oxidizing acids such as nitric acid, nitric oxides, or alternatively oxygen in combination with metal catalysts in acetic acid as a solvent can convert PE into carboxylic acids, which require costly separation from the liquid phase.^10−12,15^ Although these technologies convert PE into potentially useful products, they do not allow for conversion into basic building blocks that would enable re-synthesis of the polymer (i.e., closed-loop chemical recycling).^4,16,17^

**Scheme 1:**
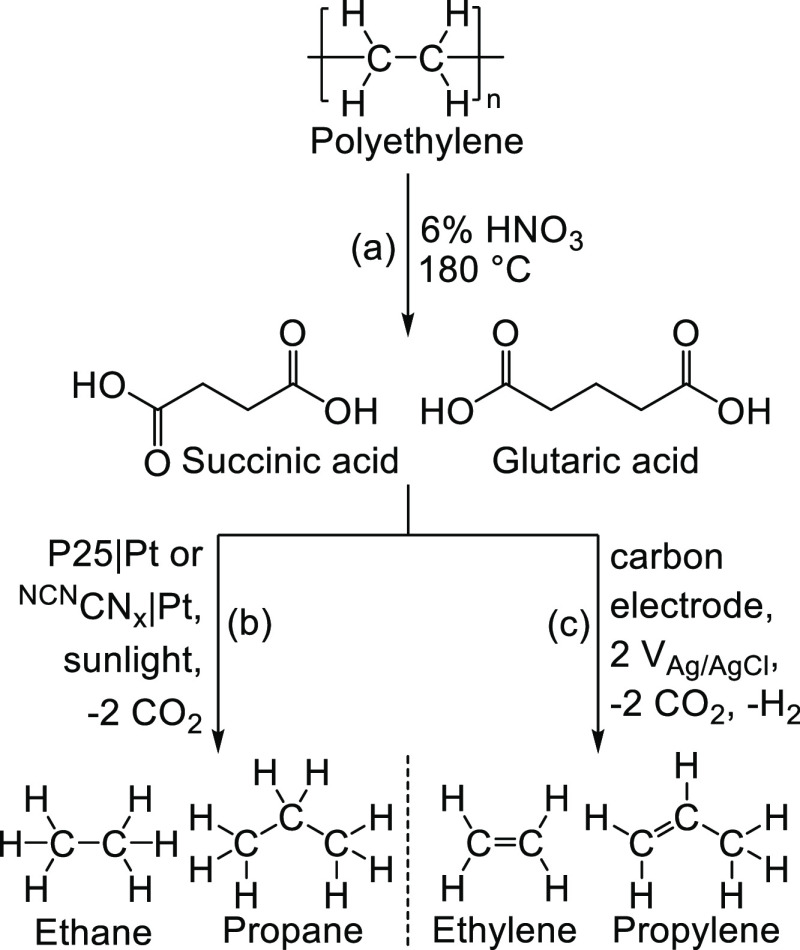
Scheme for Oxidative PE Conversion to Dicarboxylic Acids (a), Which can Subsequently be Converted into Gaseous Hydrocarbon Products via Photocatalysis to Give Alkanes (b) or Electrolysis to Produce Alkenes (c)

Here, we report a two-step process that combines an established polymer oxidative process with photocatalytic and electrocatalytic routes to convert PE via carboxylic acid intermediates into gaseous hydrocarbon products that can serve as precursors for the synthesis of new plastics or other valuable organics (e.g., ethylene oxide and vinylchloride). We show that distinct differences between the photocatalytic and electrocatalytic mechanisms lead to different product selectivities, with hydrogen being co-generated as fuel in both systems. The conversion of the carboxylic acid intermediates into gaseous products removes the requirement for complicated liquid-phase separation, and our integrated two-stage process serves as a first step toward circular chemical recycling of PE, which has the potential to significantly reduce the quantity of PE waste disposed in landfills or released into the environment (Scheme 1).

## Results and Discussion

### Oxidative PE Breakdown

The first step of the process is the batch-wise breakdown of PE (27 mg mL^–1^) (*M*_w_ 102,920 g mol^–1^, *M*_n_ 8300 g mol^–1^, PDI 12.4) to dicarboxylic acids at 180 °C for 4 h with 6 wt % HNO_3_ as the oxidant. Complete decomposition of PE is obtained with a carbon yield (moles of carbon in liquid-phase products per moles of carbon in the PE substrate) of approximately 40%, which is comparable to previous reports under microwave heating.^10^ The major products of this process include succinic (44%) and glutaric acid (22%) as determined by high-performance liquid chromatography (HPLC). The remaining products detected were acetic (21%), adipic (9%), and propanoic acid (4%) (Table S1). Longer reaction times at 180 °C did not significantly change the product distribution or carbon yield, and a lower reaction temperature (160 °C instead of 180 °C) resulted in a longer reaction time for complete PE conversion (8 h instead of 4 h). Using different types of PE that vary in molar mass and polydispersity does not result in substantially different conversion yields (Table S1). Low-density PE was used, as this material is commonly used for packaging purposes and is challenging to recycle. The proposed reaction mechanism for the oxidative conversion of PE to the dicarboxylic acids is described in the Supporting Information (Figure S1).

The PE loading was limited by the concentration of HNO_3_, with nitroxides being the reactive species that are consumed during the oxidation process. Hence, the HNO_3_ concentration decreased from 6% to ca. 0.5% after the PE decomposition [determined by ion chromatography (IC)], and a higher HNO_3_ concentration does not change the yield or product distribution significantly (Tables S1 and S2). HNO_3_ can be sourced from waste feeds found in the electronic recycling industry, and its consumption is therefore potentially beneficial for the mitigation of waste and avoidance of wastewater treatment.^18^ We have therefore also studied the PE breakdown reaction in the presence of common e-waste contaminants such as copper (10 mg mL^–1^), which gives a similar product distribution with 35% succinic acid, 24% glutaric acid, 14% adipic acid, 22% acetic acid, and 5% propanoic acid (Table S1).^18,19^

### Photocatalysis

The first oxidative step is followed by a second photocatalytic or electrocatalytic step. Photocatalysis was performed with platinum-loaded (1 wt %) P25 TiO_2_ nanoparticles (20 nm average diameter) and cyanamide-modified carbon nitride powders (^NCN^CN_*x*_, prepared from melamine at 550 °C, followed by post-synthetic modification to introduce cyanamide functional groups). P25 TiO_2_ is an archetype UV-light absorbing benchmark material for a wide range of photocatalytic reactions and, when combined with a Pt co-catalyst, has shown its proficiency for H_2_ evolution.^20−22^ Platinized carbon nitride can absorb visible light (λ < 460 nm), and the introduction of cyanamide moieties has been shown to improve the photocatalytic activity of the material due to improved charge transfer kinetics.^23,24^ A chemical reduction method was applied to load Pt nanoparticles on the two support materials. While powder X-ray diffractometry (XRD) provides the characteristic peaks for the P25 TiO_2_ and ^NCN^CN_x_ support materials, transmission electron microscopy (TEM) images show the deposition of Pt nanoparticles (3–15 nm) on the photocatalyst surface (Figures S2 and S3).

**Figure 1:**
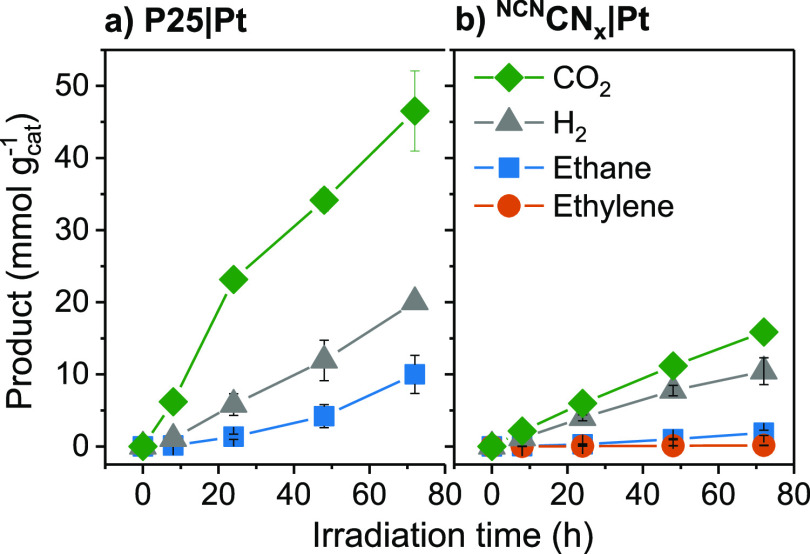
Product yields of photocatalytic experiments with (a) P25|Pt and (b) ^NCN^CN_x_|Pt. Conditions: AM1.5G, 100 mW cm^–2^, 25 °C, 2 mg mL^–1^ photocatalyst, and 2 mL of 10 mg mL^–1^ succinic acid in 0.1 M HNO_3_ set to pH 4.

The photocatalytic experiments were initially performed in sealed glass photoreactor vials (2 mL of reaction solution) with the primary product from PE decomposition, pure succinic acid. The photoreactor contained 2 mg mL^–1^ of the ultrasonicated photocatalyst (P25|Pt or ^NCN^CN_x_|Pt) and 10 mg mL^–1^ succinic acid in 0.1 M HNO_3_ under a N_2_ atmosphere and was irradiated for 24 h with simulated solar light (AM1.5G, 100 mW cm^–2^, 25 °C). The optimal performance was achieved at pH 4 (pH adjusted with NaOH; Figure S4, Table S3). The main hydrocarbon product for both P25|Pt and ^NCN^CN_x_|Pt was ethane at 56.3 and 7.2 μmol g_cat_^–1^ h^–1^ being determined by gas chromatography (GC), respectively (Figure 1 and Table S4). Ethylene was detected as a secondary product (1.3 μmol g_cat_^–1^ h^–1^) only for ^NCN^CN_x_|Pt. Significant amounts of hydrogen were also produced over both catalysts (242 μmol g_cat_^–1^ h^–1^ for P25|Pt and 137 μmol g_cat_^–1^ h^–1^ for ^NCN^CN_x_|Pt), and the decarboxylation reaction yields CO_2_ as a side product (832 μmol g_cat_^–1^ h^–1^ for P25|Pt and 231 μmol g_cat_^–1^ h^–1^ for ^NCN^CN_x_|Pt). In the aqueous phase, the intermediate product propanoic acid (964.7 μmol g_cat_^–1^ h^–1^ for P25| Pt and 176.7 μmol g_cat_^–1^ h^–1^ for ^NCN^CN_x_|Pt) and minor product adipic acid (23.5 and 25.2 μmol g_cat_^–1^ h^–1^ for P25|Pt and ^NCN^CN_x_|Pt, respectively) were detected by HPLC. Product formation and succinic acid conversion over time were verified for both photocatalysts after 72 h (Figure 1, Table S5).

The following mechanism is proposed for the photocatalytic process (Figure S5). Photoexcitation generates electron–hole pairs in the photocatalyst. The hole drives the oxidative decarboxylation of succinic acid to yield propanoic acid as an intermediate in a photocatalytic Kolbe-type reaction.^25^ In this process, the intermediate radical is quenched by H_ads_/H_2_ formed by the reductive half reaction from the excited electron and acidic water.^26^ The subsequent decarboxylation of the propanoic acid intermediate by the same mechanism yields ethane, which was confirmed using pure propanoic acid as a substrate in the photocatalytic reaction (ethane formation from propanoic acid: 338 μmol g_cat_^–1^ h^–1^ for P25|Pt and 219.5 μmol g_cat_^–1^ h^–1^ for ^NCN^CN_x_|Pt; Table S4). The decarboxylation reaction will release CO_2_, which is a gaseous byproduct in this reaction. Adipic acid can be formed as another side product upon dimerization of the intermediate radical. Ethylene can be generated upon double decarboxylation without dimerization and reduction by hydrogen. Ethylene was only formed with ^NCN^CN_x_|Pt and not with P25|Pt, which indicates that the transfer of the adsorbed hydrogen to the intermediate radical is more effective on the TiO_2_ catalyst. The hydrogen transfer on the ^NCN^CN_x_|Pt catalyst could be improved when pure H_2_ was used as the reaction atmosphere, increasing the ethane yield by approximately 35% (7.2 ± 0.9 for N_2_ compared to 9.7 ± 0.3 μmol g_cat_^–1^ h^–1^ for H_2_), while the ethylene yield remained constant (Table S4). For P25|Pt, the difference in ethane formation was not pronounced between N_2_ and H_2_ atmospheres, as the TiO_2_ surface already appears to efficiently transfer the in situ produced H_ads_/H_2_ to the radical intermediates.

Photocatalysis with ^13^C-labeled succinic acid and eitherP25|Ptor ^NCN^CN_x_|Pt revealed, using ^1^H-nuclear magnetic resonance (^1^H NMR) spectroscopy, that the evolved ethane originated from the succinic acid (Figure S6). Control experiments without a photocatalyst, light, or succinic acid did not yield any products. Without a co-catalyst (P25 or ^NCN^CN_x_ without Pt), lower quantities of ethane (1.6 μmol g_cat_^–1^ h^–1^ for P25 and 0.3 μmol g_cat_^–1^ h^–1^ for ^NCN^CN_x_) (Table S4) and higher amounts of ethylene were detected (ethylene/ethane ratio increases for carbon nitride catalysts from 1:6 for ^NCN^CN_x_|Pt to 1.1:1 for blank ^NCN^CN_x_). If Pt was replaced with MoS_2_ as an alternative co-catalyst, ethane was again the main product (1.3 μmol g_cat_^–1^ h^–1^ for P25|MoS_2_ and 3.5 μmol g_cat_^–1^ h^–1^ for ^NCN^CN_x_|MoS_2_). The evolution of H_2_ was suppressed by adding [CoCl(NH_3_)_5_]Cl_2_ as an electron scavenger,^27^ causing a sharp decline in the ethane production rate (no ethane detected for P25|Pt and 1.4 μmol g_cat_^–1^ h^–1^ for ^NCN^CN_x_|Pt) and a significant increase in the absolute ethylene production (0.5 μmol g_cat_^–1^ h^–1^ for P25|Pt and 7.1 μmol g_cat_^–1^ h^–1^ for ^NCN^CN_x_|Pt) (Table S4). These experiments support the role of hydrogen in ethane production. External quantum yields (EQYs) of 0.42% at λ = 360 nm for P25|Pt and 0.093% at λ = 400 nm for ^NCN^CN_x_|Pt were obtained, showing that ^NCN^CN_x_|Pt remains active under visible light irradiation (Table S6).

The double decarboxylation of succinic acid is a two-electron process and would yield two equivalents of H_ads_, which could potentially react with the intermediate radicals to form ethane. Ethylene formation causes the concomitant production of H_2_ gas. Nevertheless, the amount of H_2_ generated in the system exceeds that expected from the ethylene formation pathway, and some of the succinic acid is therefore likely to be oxidized completely to CO_2_ (CO_2_ was detected by GC; see Tables S4 and S5). This is consistent with our findings (Figure 1), where the amount of CO_2_ is higher than that expected if it would be solely sourced from the decarboxylation and previous reports on the mineralization of succinic acid over a variety of photocatalysts.^28−30^

Beside succinic acid, glutaric acid was also formed from the oxidative decomposition reaction of PE in the first step of the process. Photocatalysis with pure glutaric acid (11 mg mL^–1^ glutaric acid, 2 mL solution, 0.1 M HNO_3_, pH adjusted to pH 4) using P25|Pt and ^NCN^CN_x_|Pt resulted in propane (17.1 μmol g_cat_^–1^ h^–1^ for P25|Pt and 4.9 μmol g_cat_^–1^ h^–1^ for ^NCN^CN_x_|Pt) and propylene (0.04 and 0.14 μmol g_cat_^–1^ h^–1^ for P25|Pt and ^NCN^CN_x_|Pt, respectively) with butyric acid as an intermediate species (Table S7). Both primary products of the PE decomposition process could thus be readily converted by photocatalysis to gaseous hydrocarbons.

Photocatalytic conversion using the actual PE decomposition solution (rather than pure succinic or glutaric acid as model substrates) was performed under optimized conditions with the P25|Pt catalyst, as it exhibited higher activity for hydrocarbon formation than the ^NCN^CN_x_|Pt material. The pure PE decomposition solution was diluted to 10:1 with water to minimize losses from reduced light absorption by the photocatalyst, due to the yellow color of the solution. The detected products were ethane and ethylene (0.25 and 0.02 mmol g_cat_^–1^, respectively) (from succinic acid) and propane and propylene (0.14 and 0.007 mmol g_cat_^–1^, respectively) (from glutaric acid) (Table S8). The overall PE to hydrocarbon yield was determined to be 1.0% (Table S8). A higher hydrogen-to-hydrocarbon ratio was found compared to the studies with pure succinic acid (ratio of H_2_/hydrocarbon formation, 15:1 for PE solution vs 2:1 for pure succinic acid) (Table S8). This observation may be explained by the formation of other compounds such as acetic acid that will be completely oxidized without the formation of hydrocarbons and also contribute to hydrogen formation, which is also reflected by the higher CO_2_ yield of 6.1% (PE to CO_2_ carbon yield) for this process.

Beside the batch reaction, we also conducted studies applying a recently reported flow setup using a photocatalyst panel,^31^ which allowed the utilization of the pure, colored PE decomposition solution, as the light absorption losses are minimized in this configuration (Figure S7). The flow setup employed a flow cell with the mounted photocatalyst panel, where the photocatalyst was deposited by dropcasting a catalyst suspension on a frosted glass sheet (25 cm^2^; Figure S8). The optimization of the dropcasting process was discussed in our previous study.^31^ The reaction solution was pumped through the flow cell, while the photocatalyst sheet was irradiated from the back. Back irradiation of the photocatalyst panel allows light to reach the photocatalyst without being absorbed by the colored reaction solution.

**Figure 2:**
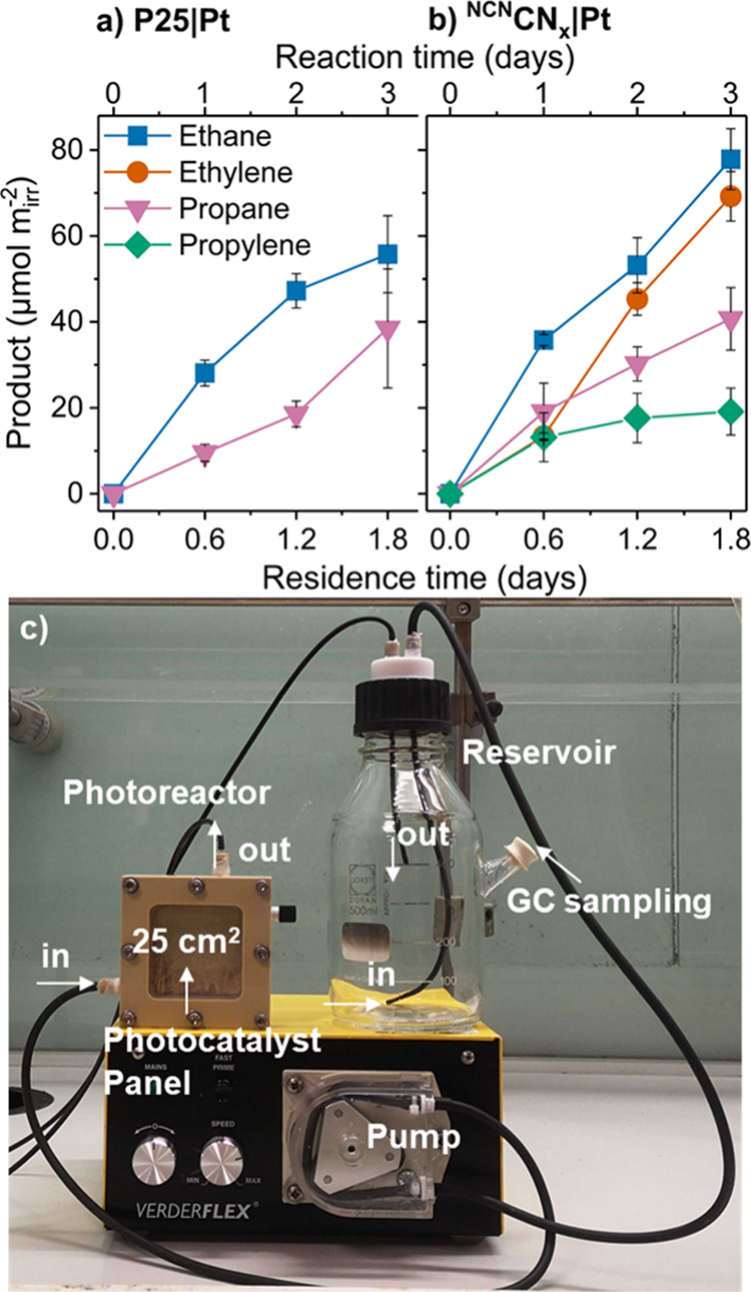
(a,b) Product yields from photocatalytic experiments using a flow setup with an irradiated area of 25 cm^2^ with (a) P25|Pt or (b) ^NCN^CN_x_|Pt deposited on glass sheets. Conditions: AM1.5G, 100 mW cm^–2^, backside irradiation, 25 °C, and 50 mL of PE decomposition solution. As the reaction time includes circulation through the reactor and the reservoir, the actual irradiation duration (residence time) is only 0.6 times the reaction time. For each equivalent of hydrocarbon, two equivalents of CO_2_ are expected to be formed (Figure S5), but the amounts were below the limit of quantification due to the large volume of the reservoir. (c) Photographic image of the photocatalytic flow setup. The PE decomposition solution (not shown in picture) is continuously pumped from a reservoir using a peristaltic pump into the photoreactor (25 cm^2^ irradiated area) before returning to the reservoir. Evolved gaseous products are sampled and analyzed by GC.

With this flow setup, a constant production of ethane (55.8 μmol m^–2^ for P25|Pt and 77.9 μmol m^–2^ for ^NCN^CN_x_|Pt), ethylene (69.2 μmol m^–2^ for ^NCN^CN_x_|Pt), propane (38.5 μmol m^–2^ for P25|Pt and 40.7 μmol m^–2^ for ^NCN^CN_x_|Pt), and propylene (19.1 μmol m^–2^ for ^NCN^CN_x_|Pt) was achieved during 72 h of reaction time (Figure 2, Table S9). The higher product yields photogenerated with ^NCN^CN_x_|Pt are likely due to the better immobilization of ^NCN^CN_x_|Pt on frosted glass. The flow system employing photocatalyst panels illustrates that even the higher concentrated PE breakdown solution can be directly converted via photocatalysis, while the challenge that colored reaction solutions pose for photocatalysis can be overcome.

### Electrocatalysis

The oxidative potential needed to drive the decarboxylation reaction can in principle also be provided electrochemically.^32^ Hence, different types of electrodes were first studied for their suitability for the conversion of succinic acid as the model substrate. The initial electrocatalytic screening was performed in a three-electrode setup with carbon paper, graphite rod, or fluorine-doped tin oxide (FTO)-coated glass as the working electrode, Pt foil as the counter electrode, and a single-junction Ag/AgCl_(sat. NaCl)_ reference electrode (reaction solution: 24 mL of 10 mg mL^–1^ succinic acid solution in 0.1 M HNO_3_, set to pH 4 with NaOH, 25 °C, single-compartment cell).

The voltammetric screening revealed that the reaction onset occurred at approximately 1.5 V (vs Ag/AgCl) (Figures S9, S10 and Table S10), yielding ethylene as the main product, with an optimal potential between 2 and 2.5 V (Faradaic yield: 12%, carbon paper, pH 4). An increased ethylene productivity was observed at higher pH values, giving a Faradaic yield of approximately 30% for carbon paper electrodes at pH 10 (Table S11). Alkaline conditions are beneficial as acid deprotonation facilitates the decarboxylation step (Figure S11), which gives access to a good Faradaic yield for this reaction.^33^ Oxygen evolution (determined using a fluorescence oxygen sensor) contributed only approximately 5% to the Faradaic yield. The remaining charge was consumed by the cyclic parasitic nitrate/nitrite redox reaction (Figure S12 and Table S12), which was also responsible for the relatively low Faradaic yield for H_2_ evolution at the counter electrode (see the Supporting Information for more details). A minor gaseous side product was acetylene, while adipic acid was detected in the liquid phase (see Figure S11 for the proposed reaction mechanisms). The CO_2_ derived from the decarboxylation reaction is another gaseous byproduct. Tests with ^13^C-labeled succinic acid confirm that the evolved ethylene originated from the succinic acid using ^1^H NMR spectroscopy (Figure S13). In the absence of succinic acid, no ethylene or other hydrocarbon products were detected, except for small amounts of CO_2_, indicating a slow, gradual oxidation of the carbon anode. Glutaric acid could also be converted, yielding propylene (Faradaic yield of 4%, 10.6 μmol cm_cat_^–2^ h^–1^, at 2 V vs Ag/AgCl) (Table S13).

**Figure 3:**
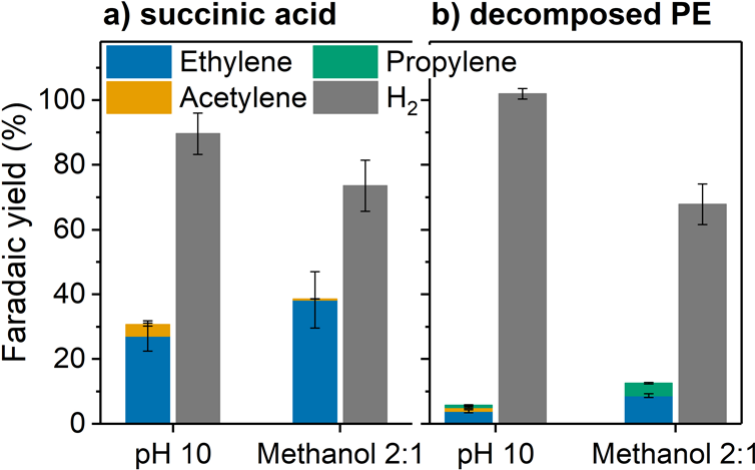
Electrolysis with (a) succinic acid solution, 3.3 mg mL^–1^, in 0.1 M aqueous HNO_3_ set to pH 10 by addition of NaOH or in 2:1 methanol/0.1 M aq. HNO_3_ (set to pH 10 with NaOH) solutions; (b) PE decomposition solution set to pH 10 or 2:1 diluted with methanol. Working electrode: carbon paper (2 cm^2^ electrode area), counter electrode: Pt foil (2 cm^2^ electrode area), applied voltage was 5 V until approximately 130 C (= 1 Faradaic equivalent) has been passed through the cell. For each equivalent of hydrocarbon, two equivalents of CO_2_ are expected to be formed from the decarboxylation reaction (Figure S11).

After identifying suitable electrochemical conditions, a two-electrode electrolyzer was assembled as a proof-of-principle device (conditions: 24 mL of electrolyte solution, carbon paper working electrode, Pt foil counter electrode, 25 °C, and single-compartment cell). Electrolysis was conducted at pH 10 (highest Faradaic yield) until 1 Faradaic equivalent has passed (1 Faradaic equivalent based on the amount of charge necessary to theoretically convert all the present substrate) in solvent mixtures of water and methanol, which is known to improve the performance of electrocatalytic decarboxylation reactions (such as the Kolbe reaction).^33,34^ Studies with succinic acid in a 2:1 mixture of methanol and 0.1 M HNO_3_ (adjusted to pH 10) with 1 Faradaic equivalent required a longer electrolysis time, presumably due to the lower conductivity of the methanol solution. Faradaic yields for ethylene of 27% under aqueous conditions (pH 10) and 38% under methanolic conditions were achieved (ethylene formation rate: 47.9 and 21.0 μmol cm_cat_^–2^ h^–1^ for pH 10 and methanolic conditions, respectively) (Figure 3a, Table S14). Under methanolic conditions, only trace amounts of acetylene were formed (compared to 3% Faradaic yield and 4.3 μmol cm_cat_^–2^ h^–1^ under aqueous pH 10 conditions). For both conditions, adipic acid was found as a side product (5.8 and 5.4 μmol cm_cat_^–2^ h^–1^ for pH 10 and methanolic conditions, respectively) (Table S14). The overoxidation of methanol itself is low and contributes a maximum of 4% to the Faradaic yield (Table S15).

After studying electrolysis of the pure organic acid intermediates, the PE decomposition solution (with a starting concentration of 27 mg mL^–1^ PE) was subsequently set to pH 10 by adding a NaOH solution and studied in pure aqueous and 2:1 MeOH/H_2_O solution. In addition to ethylene (Faradaic yield of 4% or 9% and product formation rate of 6.0 or 5.0 μmol cm_cat_^–2^ h^–1^ for pH 10 and methanolic conditions, respectively), also, propylene (from glutaric acid) (0.6% or 3.9% Faradaic yield and product formation rate of 1.1 or 2.2 μmol cm_cat_^–2^ h^–1^ for pH 10 and methanolic conditions, respectively) and butylene (originating from the adipic acid in the solution) were detected (Figure 3b, Table S16). The Faradaic yields were lower compared to those of pure succinic acid (even when adding up all gaseous products). This can be explained by the presence of longer chain diacids in the PE solution, which have been shown to be less reactive than succinic acid for the double-decarboxylation reaction.^33^ In aqueous solution, acetylene was formed (0.9% Faradaic yield, 1.9 μmol cm_cat_^–2^ h^–1^), which was not the case in methanolic solutions (Table S16). The Faradaic yield for H_2_ was slightly higher for aqueous and lower for methanolic conditions, compared with the pure succinic acid solution (Figure 3, Tables S14 and S16). Methanolic conditions give overall a higher Faradaic yield of hydrocarbons than the aqueous conditions (Figures S14 and S15).

The overall yield from PE to hydrocarbons (ethylene and propylene) is 7.6%, and a PE to CO_2_ yield of 13.5% has been calculated based on the total amount of CO_2_ detected (note that some CO_2_ may have also been produced from electrode degradation and MeOH oxidation; Table S17). On the cathode, hydrogen is being formed. To assess the overall process, also, the consumption of the auxiliary reagents (HNO_3_, methanol, and carbon electrode) was determined and is summarized in the Supporting Information (Table S15). A total of 90% of HNO_3_ was converted in the PE breakdown step, and the nitrate concentration remained constant during the electrocatalytic conversion. The carbon electrode in electrocatalysis undergoes slow but gradual degradation to CO_2_, and this process is reduced in methanolic solution. However, the presence of methanol can also cause some overoxidation to CO_2_. A combined Faradaic yield of 4% was found overall for methanol and carbon electrode overoxidation and is therefore only a minor contributor to the charge flowing in the electrolyzer.

### Comparison to Established Technologies

Finally, we compare our tandem processes with existing conversion technologies for PE waste such as pyrolysis and gasification (see Table S18 for details). Pyrolysis of plastic waste requires high temperatures of 450–600 °C and converts PE waste into a complex mixture of mainly liquid hydrocarbons (50–70% yield of C_5_–C_20+_ hydrocarbons).^35−37^ The obtained hydrocarbon mixture (often termed as syn-crude, due to its resemblance with crude oil) can be further converted into alkenes such as ethylene and propylene by steam cracking at 650–820 °C.^38,39^ The combined ethylene and propylene yield of the steam cracking process lies between 30 and 50%, giving a total estimated plastic to alkene yield of 15–35%. Byproducts formed in steam reforming include CO_2_, CO, CH_4_, C_2_H_6_, C_3_H_8_, various C_4_ and C_5_ products and a variety of aromatics (more than 10 have been described).^38−41^

Gasification of PE is conducted at 600–1000 °C (often in the presence of steam and/or air) and yields a gas mixture of CO, H_2_, CO_2_, and CH_4_, and the precise product composition depends on the process conditions (temperature, residence time, and catalyst).^5^ The syngas (CO and H_2_) produced can be subsequently converted over zeolite catalysts at approximately 400 °C into C_2_–C_4_ olefins. C_5+_ products and CH_4_ are prominent byproducts in this second process step. A combined plastic to C_2_–C_4_ alkene yield of <40% can be estimated for such a process.^42,43^

While pyrolysis and gasification have currently higher PE to alkene conversion yields (15–35% vs 7.6%), these technologies are already optimized and operate at scale, require significantly higher temperatures (600–1000 °C vs 180 °C), and yield more complex product mixtures that demand more elaborate separation procedures. Despite its currently lower yields, the reported two-step processes benefit from a significantly lower thermal energy input, yield a less-complex product mixture, and have much scope to improve yields and selectivities in further optimization and scale up. Additionally, solar and electrochemical processes can be easily decentralized, which makes them interesting for small- to middle-sized applications.

Several strategies can be employed to improve the reported processes in future development. The generated CO_2_ can be separated downstream and itself catalytically be converted to ethylene, which has been reported as an isolated electrochemical process with high Faradaic yields (>60%).^44^ Furthermore, advances in the HNO_3_-mediated decomposition by more elaborated reactor concepts or general process improvements can enhance the carbon yield of the first step. The development of catalysts with higher efficiency for the second electro-/photocatalytic steps will also improve conversion yields. These approaches may enable a significant enhancement of the overall PE to hydrocarbon yield and make the processes more commercially feasible.

## Conclusions

We report a two-step process to convert waste PE into gaseous hydrocarbons such as ethane and ethylene, which accumulate in the reaction atmosphere and can thus be easily separated from the reaction solution. The first step employs an oxidation reaction in diluted nitric acid, which can potentially be sourced from waste feeds. The obtained dicarboxylic acids, mainly succinic and glutaric acid, can then be converted to gaseous alkanes and alkenes by photo- or electrocatalytic decarboxylation reactions that also yield H_2_ and CO_2_ as gaseous byproducts. The chosen pathway affected the product selectivity: photocatalysis mainly yields alkanes such as ethane and propane, whereas electrocatalysis produces mainly alkenes such as ethylene and propylene, due to the distinct reaction mechanisms. This process provides an entry point to closed-loop chemical recycling of plastic waste by converting it to valuable, easily separable gaseous hydrocarbon products, with a plastic to hydrocarbon yield (ethylene and propylene) of 7.6%.

## Experimental Section

### Material Synthesis

Carbon nitride (CN_x_) was prepared following a literature procedure.^23^ Briefly, 2 g of melamine was heated in a covered crucible to 550 °C for 4 h (heating ramp of 5 °C min^–1^). For post-synthetic modification, the as-obtained CN_x_ was mixed and ground with KSCN (weight ratio of 1:2 for CN_x_/KSCN) using a mortar and pestle.^23^ The mixture was heated under an Ar atmosphere to 400 °C for 1 h followed by a temperature increase to 500 °C (ramp rate 30 °C min^–1^) and holding at this temperature for 30 min. After cooling to room temperature, the resulting ^NCN^CN_x_ material was thoroughly washed with water and dried at 80 °C overnight. Characterization details can be found in a previous report.^24^ P25 TiO_2_ nanoparticles (Evonik, anatase/rutile, 21 nm) were used as received.

For platinum loading, a literature procedure was adapted:^45^ 150 mg of the support material (^NCN^CN_x_ or P25) was dispersed in 10 mL of H_2_O with sonication for 30 min. Then, 0.29 g of trisodium citrate dihydrate was added, and the dispersion was further sonicated for 30 min before 42 μL of H_2_PtCl_6_ solution (8 wt % in H_2_O) was added. After further sonication for 30 min, the mixture was stirred with a magnetic stirrer, and a total of 5 mg of NaBH_4_ dissolved in 1 mL of H_2_O was added. After stirring for 30 min, the material was isolated via centrifugation, washed with H_2_O, and dried at 80 °C overnight.

The MoS_2_ deposition was conducted in situ by adding an aqueous solution of ammonium tetrathiomolybdate to the reaction solution (final MoS_2_ loading of 2 wt %) and irradiation with artificial sunlight using a solar light simulator (Newport Oriel, 100 mW cm^–2^) equipped with an AM1.5G filter, following a published procedure.^46^

### Material Characterization

TEM was conducted on a Thermo Scientific (FEI) Talos F200X G2 TEM. All samples were dropcast on carbon-coated Cu grids (300 mesh). X-ray powder diffractometry was conducted on a PANalytical Empyrean Series 2 instrument using Cu Kα irradiation.

### Plastic Decomposition

A total of 300 mg of PE was dispersed in 11 mL of 6% HNO_3_ in a Teflon pot and sealed in an autoclave. Among a series of different conditions, the most effective treatment, leading to complete decomposition of the PE, was heating to a temperature of 180 °C and holding for 4 h. Longer reaction times did not significantly change the product distribution and yield. At lower temperatures, significantly longer reaction times would have been necessary to obtain the same amount of PE conversion. The obtained solution was clear and yellow-colored and is denoted as “PE decomposition solution”. To study the influence of contaminants, 10 mg mL^–1^ copper (as Cu(NO_3_)_2_·6H_2_O) was added into the Teflon pot before sealing the autoclave.

Calculation of carbon yield
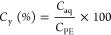
*C*_y_ = carbon yield in %, *C*_aq_ = sum of all detected products in the aqueous phase (in mol) multiplied with their number of carbon atoms (e.g., 4 for succinic acid), *C*_PE_ = total carbon atoms in utilized PE (in mol; e.g., 300 mg of PE = 21.4 mmol carbon).

### Photocatalysis in Batch Mode

The catalyst powder (Pt-loaded or unloaded P25 or ^NCN^CN_x_; 4 mg) was dispersed by sonication in 2 mL of reaction solution (pH was measured with a Mettler Toledo Seven Easy pH electrode) (MoS_2_ was photodeposited in situ by adding 20 μL of aqueous 10 mg mL^–1^ (NH_4_)_2_MoS_4_ solution into the photoreactor vials). For control experiments, 10 mg of [CoCl(NH_3_)_5_]Cl_2_ was added. The prepared samples were added to Pyrex glass photoreactor vials (internal volume of 7.91 mL) and capped with rubber septa. After briefly vortexing, the samples were purged with N_2_ (containing 2% CH_4_ as an internal standard and leakage control in GC analysis) or H_2_ at ambient pressure for 10 min. The samples were then irradiated using a calibrated solar light simulator (Newport Oriel, 100 mW cm^–2^) equipped with an AM1.5G filter and a water filter to remove infrared radiation. All samples were stirred at 600 rpm and kept at 25 °C during irradiation. Product generation was monitored by periodically analyzing samples of the reactor head space gas (50 μL) by GC (see below). For tests longer than 24 h, the overpressure was reduced by enlarging the headspace.

Calculation of carbon mass balance
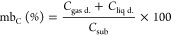
mb_C_ = mass balance of carbon in %, *C*_gas d_ = sum of all detected products deteced in the gas phase (in mol) multiplied with their number of carbon atoms (e.g., 2 for ethylene), *C*_liq d_ = sum of all detected products deteced in the liquid phase (in mol) multiplied with their number of carbon atoms (e.g., 4 for succinic acid), and *C*_sub_ = total carbon atoms in the utilized substrate (in mol; e.g., 20 mg of succinic acid = 0.677 mmol carbon).

### External Quantum Yield

The photocatalyst (Pt-loaded P25 or ^NCN^CN_x_; 3 mg) and 2 mL succinic acid solution (10 mg mL^–1^, set to pH 4) were added to a quartz cuvette (path length 1 cm), which was then sealed with a rubber septum. The sample was purged with N_2_ containing 2% CH_4_ for 10 min. While stirring, the sample was irradiated using a Xe lamp (LOT LSH302) fitted with a monochromator (LOT MSH300) focused at a single wavelength of λ = 360 nm (for P25|Pt) or 400 nm (for ^NCN^CN_x_|Pt) (accurate to a full width at half maximum of 5 nm). The light intensity was adjusted to ∼1000 μW cm^–2^, as measured with a power meter (ILT 1400, International Light Technologies). The cuvette was irradiated across an area of 0.28 cm^2^. The evolved headspace gas was analyzed by GC, and the EQY (%) was calculated via
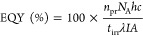
where *n*_pr_ is the amount of ethane or ethylene generated (mol), *N*_A_ is Avogadro’s constant (mol^–1^), *h* is Planck’s constant (J s), *c* is the speed of light (m s^–1^), *t*_irr_ is the irradiation time (s), λ is the wavelength (m), *I* is the light intensity (W m^–2^), and *A* is the irradiated area (m^2^).

### Photocatalysis in Flow

The photocatalytic flow setup and the preparation of the utilized catalyst glass sheets were already described elsewhere.^31^ The first step is the preparation of the photocatalyst sheets, employing frosted glass (5 × 5 cm^2^) that was cleaned by sonication with isopropyl alcohol and acetone for 15 min each, followed by drying under a N_2_ stream. ^NCN^CN_x_ |Pt or P25|Pt was dispersed in ethanol (20 mg mL^–1^) by ultrasonication (10 min, pulses of 30 s at 100% amplitude followed by 5 s pauses), and 1 vol % Nafion solution (5 wt %) was added to the resulting mixture. A total of 16 μL cm^–2^ dispersion was dropcast onto clean frosted glass and allowed to dry at ambient temperature (typically for 2–10 min) before the addition of subsequent layers (total amount: six layers or 1.92 mg_cat_ cm^–2^). The prepared photocatalyst panels were then annealed at 80 °C overnight in air. The as-prepared photocatalyst sheets were mounted in the flow photoreactor.

A reservoir (500 mL) was filled with a substrate mixture (50 mL of PE decomposition solution) and connected to the peristaltic pump and photoreactor (internal volume of 5 × 5 × 1.2 cm^3^, 30 mL) using Viton tubing (inner diameter of 1.6 mm). While continuously circulating the mixture between the reservoir and photoreactor at a high flow rate (10–20 mL min^–1^), the reservoir was purged with N_2_ (containing 2% CH_4_ for leakage control) at ambient pressure for 1 h. The photoreactor was then irradiated from the back (to avoid light passing through the colored reaction solution) using a solar light simulator (AM1.5G, 100 mW cm^–2^, LOT-Quantum Design) under a flow rate of 2 mL min^–1^. Reaction products were monitored by periodical manual sampling and analyzing aliquots of the reservoir headspace (50 μL) by GC. For HPLC analysis, the reaction solution was manually sampled (0.5 mL), and the obtained samples were diluted with H_2_O before measurements.

### Electrocatalysis

All electrochemical experiments were conducted with an Ivium CompactStat potentiostat. A total of 24 mL of the particular reaction solution (containing succinic acid, glutaric acid, or the PE decomposition solution) was purged with N_2_ (with 2% CH_4_ as internal standard) for 20 min before conducting the experiments. A three-electrode setup consisting of an Ag/AgCl (sat. NaCl) (BasiMW-2030) reference electrode, a platinum foil counter electrode, and a working electrode (either carbon paper with 2 cm^2^ electrode area, graphite rod with 2 cm^2^ electrode area, or FTO-coated glass with 1 cm^2^ electrode area) was used for initial screening.

For longer time electrolysis studies, the reference electrode was removed, and a two-electrode setup was used with carbon paper as the working electrode and Pt foil as the counter electrode. In this case, the electrochemical cell was equipped with additional headspace volume (130 mL glass bubble), to accommodate the larger amounts of gaseous products. The applied voltage for the two-electrode studies was set to 5 V, allowing current densities of approximately 20–30 mA cm^–2^ for tests under aqueous conditions. Comparable current densities were reached for the three-electrode setup at set potentials between 2 and 2.5 V versus Ag/AgCl (sat. NaCl). All potentials for the three-electrode electrochemistry are stated versus Ag/AgCl (sat. NaCl). Reaction products were monitored by manual sampling and analyzing aliquots of the reaction vessel headspace (50 μL) by GC at the end of the reaction. Samples for HPLC analysis (0.5 mL) were also sampled manually and analyzed without further processing.

### Product Analysis

The accumulated hydrocarbon products, CO_2_, and H_2_ were measured in the headspace using an Agilent 7890A gas chromatograph equipped with a flame ionization detector and thermal conductivity detector. The splitless injection mode was applied with an inlet temperature of 120 °C, and a PLOT-MS 5A Molsieve column and a HP PLOT Q column were used for product separation, with N_2_ as the carrier gas and a constant oven temperature of 50 °C and a pressure of 16.0 psi. Methane (2% in N_2_) was used as an internal standard and to control any leakage of the reaction vessels. Gas calibration mixtures containing a known amount of the particular product were utilized to quantify the detected amount of the products (Figure S16).

^1^H NMR spectroscopy was used to analyze isotopically-labelled gaseous products by transferring the reaction atmosphere into an evacuated Young NMR tube (thicker glass) with *d*_6_-benzene as the solvent. NMR spectra were collected with a Bruker 400 MHz Neo Prodigy spectrometer. The O_2_ evolution was traced using a NeoFox-GT fluorometer and Fospor-R fluorescence oxygen sensor probe from Ocean Optics.

HPLC separations were conducted with a Phenomenex Rezex 8% Ca^2+^ column at 75 °C column temperature. Samples were analyzed in the isocratic flow mode (flow rate of 0.025 M H_2_SO_4_ in water of 0.5 mL min^–1^) using a Waters Breeze system equipped with refractive index (RID-2414) and diode array UV–vis (λ = 254 nm) detectors. To identify particular substances in the reaction samples, retention times were compared to those of authentic samples (Figure S17). Calibration was conducted with external standards. IC was performed with a 882 Metrohm Compact IC Plus using 3.2 mM Na_2_CO_3_ and 1 mM NaHCO_3_ as an eluent.

## Supporting Information Available

Raw data related to this article are available at the University of Cambridge data repository: https://doi.org/10.17863/CAM.71937. The Supporting Information is available free of charge at https://pubs.acs.org/doi/10.1021/acscatal.1c02133.

Information regarding reagents, oxygen determination and nitrate redox cycle, calculation of auxiliary reactant conversion as well as Figures S1–S17 and Tables S1–S18 (PDF)
