# Supplementary material for: Conversion of Polyethylene Waste into Gaseous Hydrocarbons via Integrated Tandem Chemical–Photo/Electrocatalytic Processes
Source: ACS Catal. 2021 Jul 9;11(15):9159–67. doi: 10.1021/acscatal.1c02133 (PMC8353629; doi:10.1021/acscatal.1c02133)
Supplement: Supplementary file 1 — cs1c02133_si_001.pdf [file cs1c02133_si_001.pdf]

Supporting Information

Conversion of polyethylene waste into gaseous hydrocarbons via integrated tandem chemical – photo/electrocatalytic processes

Christian M. Pichler, Subhajit Bhattacharjee, Motiar Rahaman, Taylor Uekert and Erwin Reisner*

Yusuf Hamied Department of Chemistry, University of Cambridge, Lensfield Road, CB2 1EW Cambridge, UK; Corresponding author email: reisner@ch.cam.ac.uk

Reagents

Melamine, chloroplatinic acid, trisodium citrate dihydrate, sodium borohydride, succinic acid, ^{13}C labelled succinic acid, glutaric acid, propanoic acid, adipic acid, butyric acid, pentaaminechlorocobalt(III) chloride, ammonium tetrathiomolybdate, FTO-coated glass, graphite rods (diameter 2 mm), 5 wt.% Nafion solution (in lower aliphatic alcohols and water) were purchased from Sigma Aldrich. Potassium thiocyanate was purchased from Fisher Scientific. Low density polyethylene powders were purchased from Goodfellows Ltd (Exxonmobil LD 654) (M_w 102920 g mol^{-1}, M_n 8300 g mol^{-1}, PDI 12.4), which was used as standard substrate or Sigma Aldrich (M_w 35000 g mol^{-1}, M_n 7700 g mol^{-1}, PDI 4.5) for comparison. P25 (Surface area: 50 m^2 g^{-1}) was a gift from Evonik. Toray TGP-H-60 carbon paper (uncoated) was purchased from Alfa Aesar. All chemicals were used as received.

Oxygen determination and nitrate redox cycle

It has been shown that the oxygen evolution reaction only contributes around 5% to the Faradaic yield, in addition to the approximately 30% from the ethylene formation. The remaining charge is used in the parasitic nitrate/nitrite redox cycle (or other competing processes). In this nitrate/nitrite redox cycle, nitrate is being reduced at the cathode to nitrite or other species such as ammonium, but the products will be reoxidized at the anode (Figure S12).[1] This cycle has already been shown to interfere with the related Kolbe reaction.[2]

To test the effect of the nitrate/nitrite redox cycle, the suppression of oxygen evolution shall be demonstrated in the presence of nitrate. For comparison reasons, a 0.05 M NaOH aqueous solution (without the presence of nitrate) is electrolyzed, giving a Faradaic yield of 86% for O_2. If nitrate is present and a 0.1 M HNO_3 aqueous solution (set to pH 10 by NaOH) undergoes the same reaction, the Faradaic yield of oxygen evolution is only < 1%. This is a strong indication, that nitrate is suppressing the oxygen evolution, but undergoing a redox cycle itself. No other substrate is present, that could undergo anodic reactions, unless nitrate is reduced to intermediates. The reduction of nitrite has been reported to take place under the utilized reaction conditions.[3,4] Hence, the low faradaic yield for oxygen evolution for the succinic acid solution (0.1 M HNO_3, pH10) can be expected. See Table S12.

Calculation of auxiliary reactant conversion

HNO_3: Consumed in the PE decomposition step

$$C_{HNO3} = \frac{n_{HNO3\ start} - n_{HNO3\ end}}{w_{PE}}$$

C_{HNO3}… Consumption of HNO_3 in mol g^{-1} PE

$n_{HNO3\ start}$… number of mol HNO_3 before the PE decomposition

$n_{HNO3\ end}$… number of mol HNO_3 after the PE decomposition

w_{PE}… weight of PE in g decomposed

Methanol: Consumed during electrocatalysis

$$C_{MeOH} = \frac{FY_{side} * n_{gas}}{FY_{gas} * n_{side}}$$

C_{MeOH}… Consumption of MeOH in mol mol^{-1} formed gaseous product

FY_{side}… Faradaic yield for MeOH oxidation (assumed to form CO_2)

FY_{gas}… Faradaic yield for formation of gaseous products

n_{side} … number of electrons utilized in MeOH oxidation (assumed to form CO_2)

n_{gas} … number of electrons utilized in formation of gaseous products

Carbon electrode: Consumed during electrocatalysis

$$C_{carb} = \frac{FY_{side} * n_{gas}}{FY_{gas} * n_{side}} * M_{carb}$$

C_{carb}… Consumption of carbon electrode material in g mol^{-1} formed gaseous product

FY_{side}… Faradaic yield for carbon electrode (assumed to form CO_2)

FY_{gas}… Faradaic yield for formation of gaseous products

n_{side} … number of electrons utilized in carbon oxidation (assumed to form CO_2)

n_{gas} … number of electrons utilized in formation of gaseous products

M_{carb}… molar mass of carbon

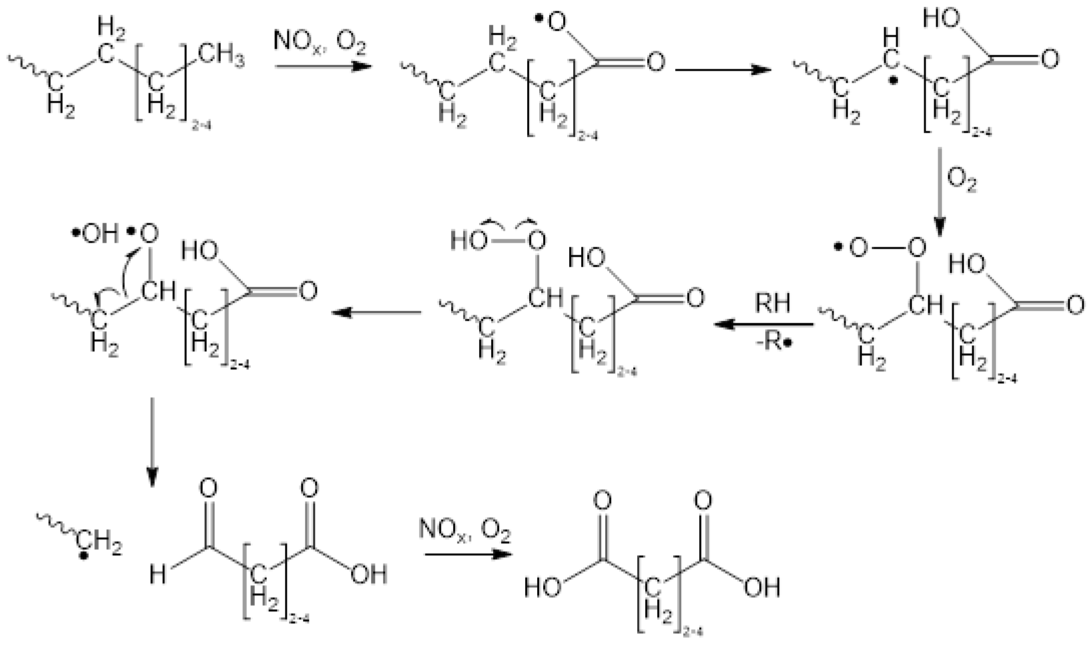

Figure S1. Proposed mechanism for oxidative PE decomposition.[5,6]

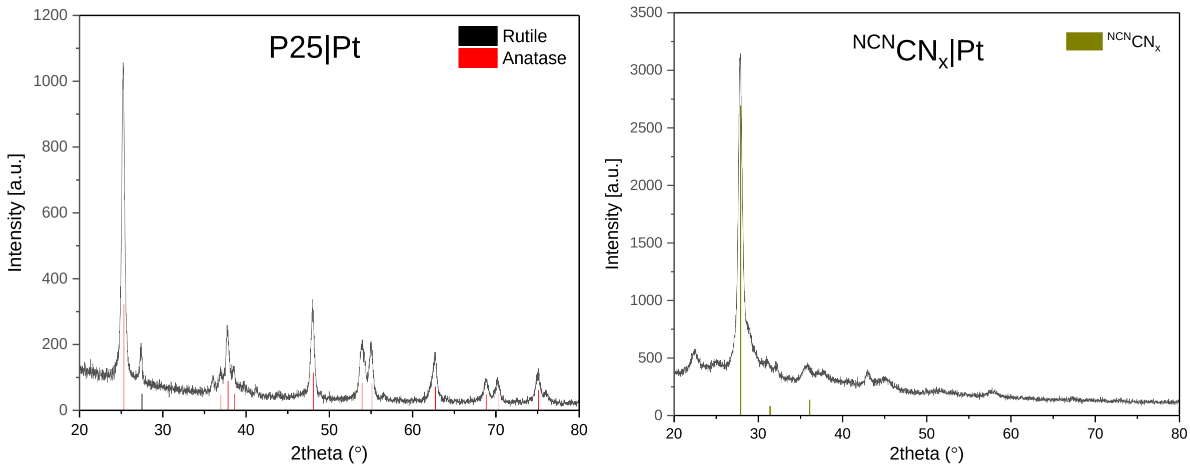

Figure S2. Powder XRD patterns of P25|Pt and $^{NCN}CN_x$|Pt. The diffractograms show the expected TiO_2 phases for P25, reflections for rutile (black) and anatase (red) and the carbon nitride (yellow) can be identified.

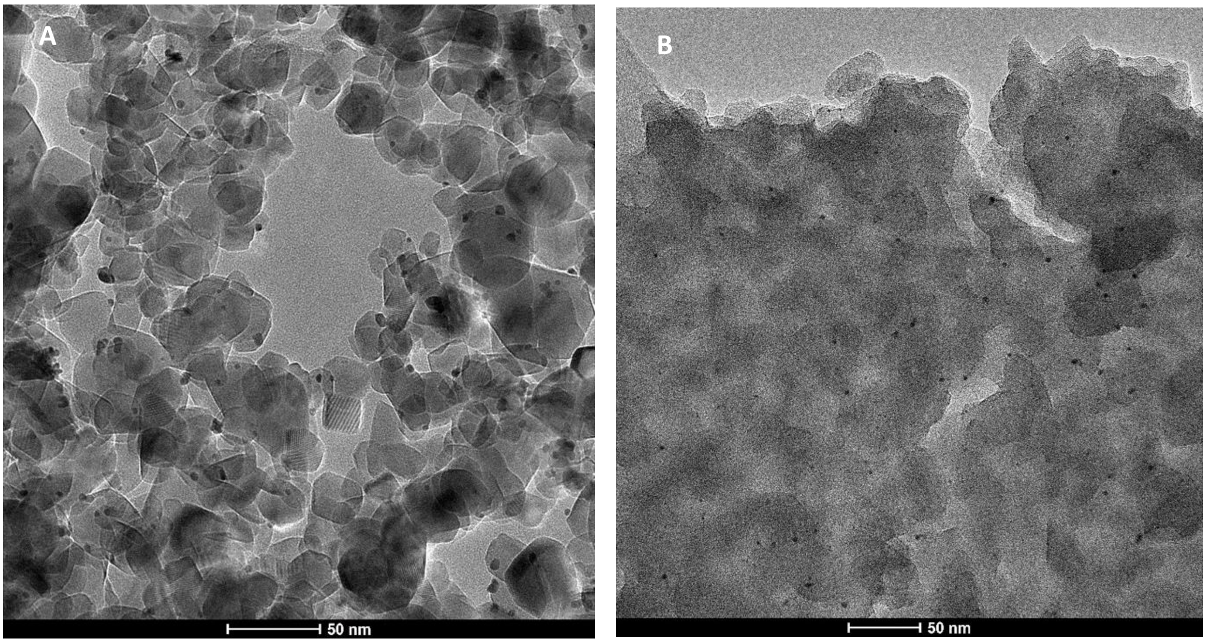

Figure S3. TEM images of P25|Pt (A) and $^{NCN}CN_x$|Pt (B). Pt particles have a size of approximately 5-15 nm on TiO_2, whereas the Pt particles are slightly smaller with approximately 3-8 nm on $^{NCN}CN_x$. (Operating voltage: 250 kV, bright field mode)

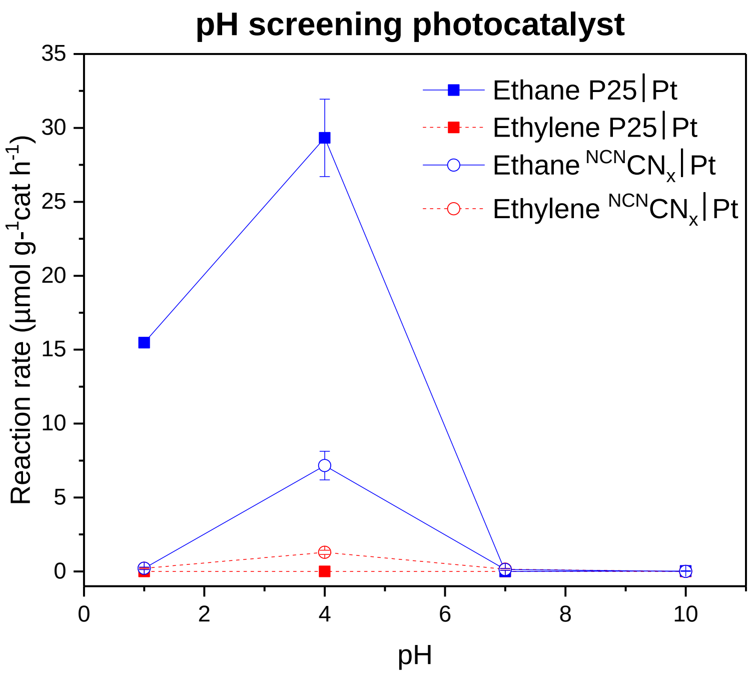

Figure S4. Photocatalytic conversion of succinic acid at different pH values using AM1.5G, 100 mW cm^{-2} irradiation at 25 °C (4 mg catalyst, 2mL of 10mg mL^{-1} succinic acid solution in 0.1 M HNO_3 set to different pH values with 10 M NaOH, 24 h irradiation time)

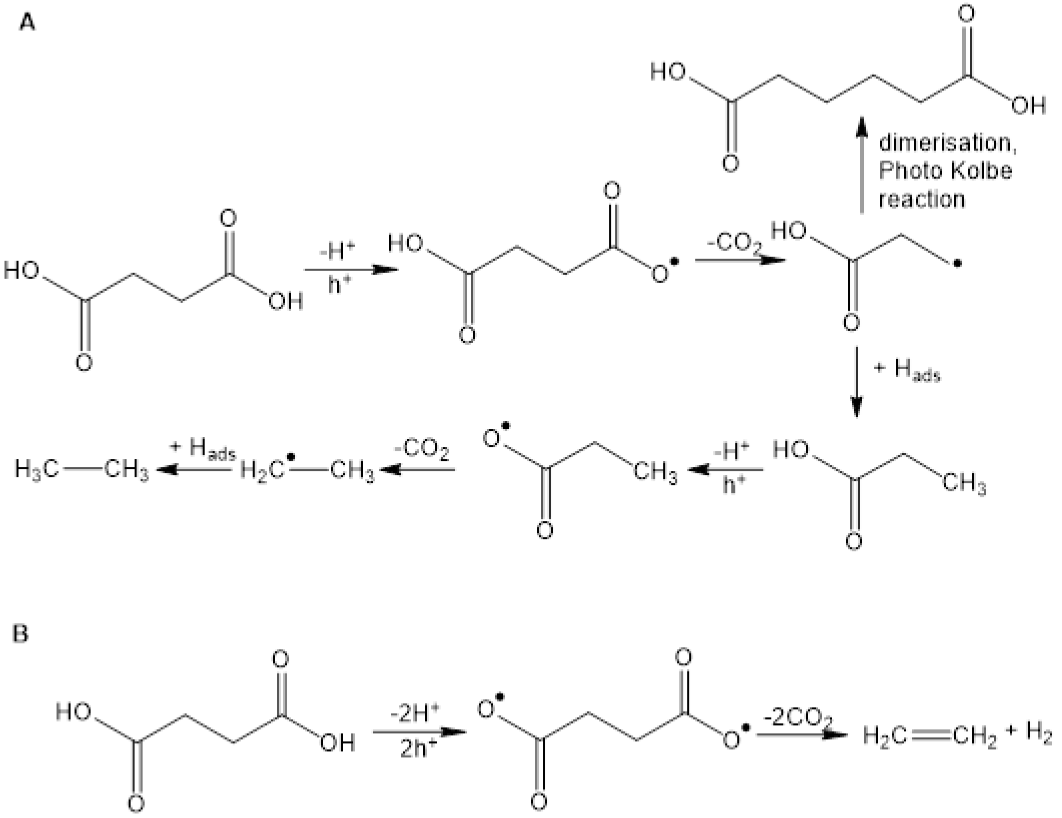

Figure S5. Proposed mechanisms for (A) photocatalytic decarboxylation with hydrogen transfer, (B) double photocatalytic decarboxylation without hydrogen transfer (taking place mainly on the $^{NCN}CN_x$|Pt catalyst).

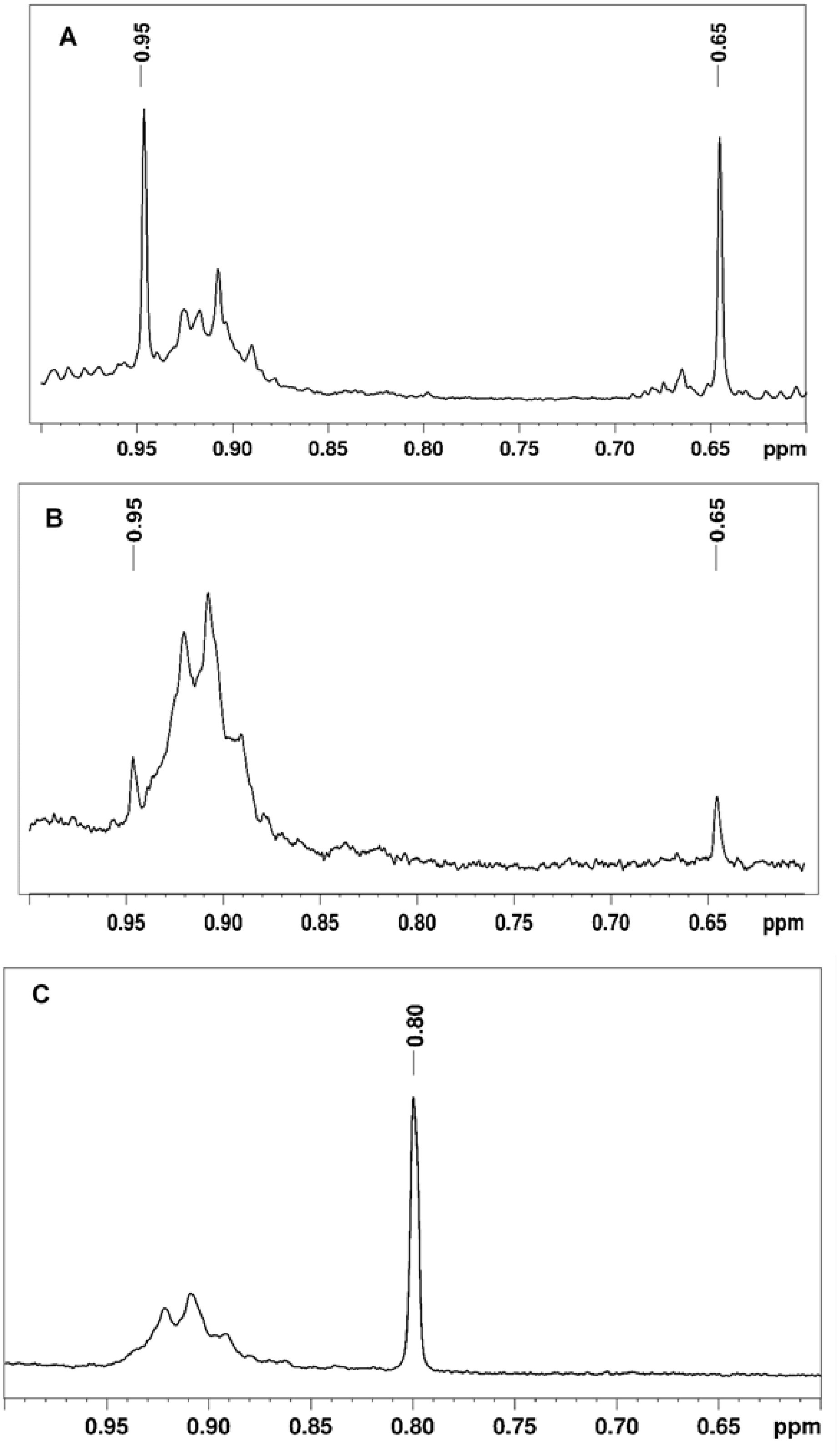

Figure S6. ^{1}H-NMR spectra of ethane generated by photocatalytic experiments using AM1.5G, 100 mW cm^{-2} irradiation at 25 °C, with 4 mg P25|Pt or $^{NCN}CN_x$|Pt, 2 mL of 10 mg mL^{-1} succinic acid solution (either ^{13}C labelled or not) in 0.1 M HNO_3 set to pH 4, (A), ^{13}C labelled succinic acid with P25|Pt catalyst, (B) ^{13}C labelled succinic acid with $^{NCN}CN_x$|Pt catalyst, (C) unlabeled succinic acid with P25|Pt catalyst. The ^{13}C labelled succinic acid yields ^{13}C labelled ethane, causing a splitting of the proton-signal at 0.8 ppm (observed with the unlabeled succinic acid in (C)) into two signals at 0.64 and 0.94 ppm (A and B), coupling constant J=120 Hz.

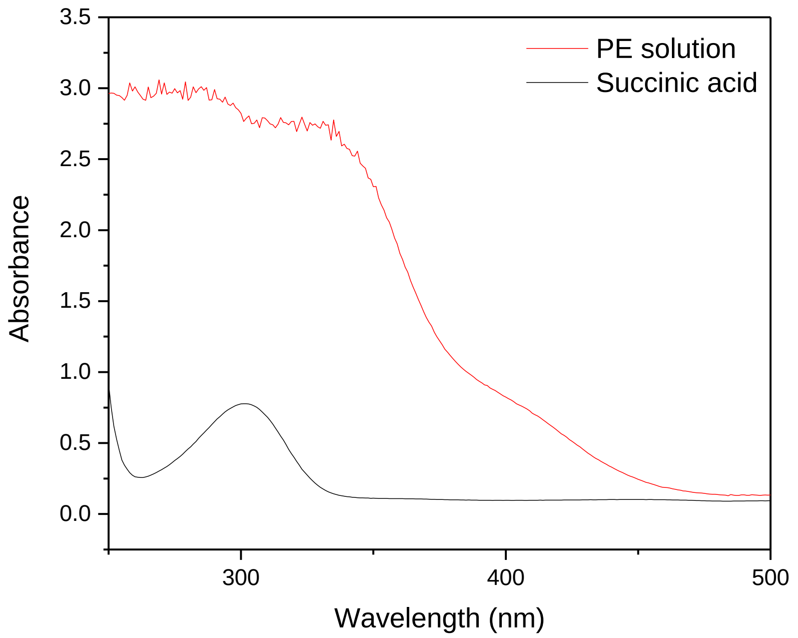

Figure S7. UV-VIS absorption spectra of 10mg mL^{-1} succinic acid in 0.1 M HNO_3 (black) and PE decomposition solution (red), 25 °C.

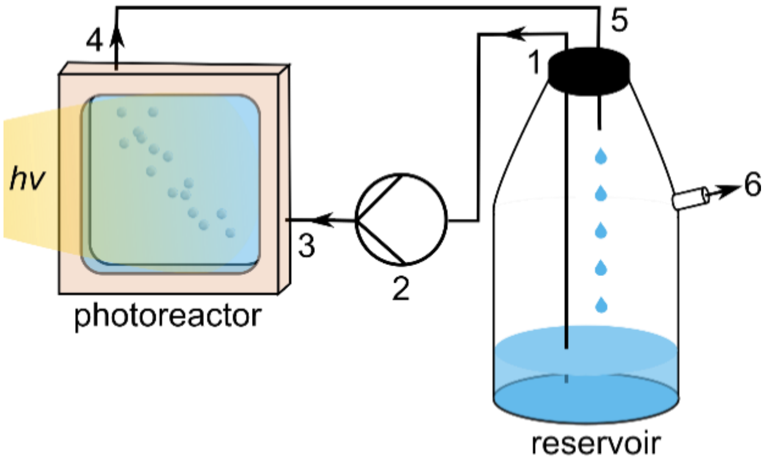

Figure S8. Flow setup with an irradiated area of 25 cm^2, AM1.5G, 100 mW cm^{-2} backside irradiation at 25 °C, with P25|Pt or $^{NCN}CN_x$|Pt deposited on glass sheets, 50 mL of PE decomposition solution. The photoreforming solution is continuously pumped out of a reservoir (1) by a peristaltic pump (2) through the inlet (3) and outlet (4) of the photoreactor before returning to the reservoir (5). Evolved H_2 is sampled from the reservoir outlet (6) and analyzed by GC. Figure adapted from reference 7.

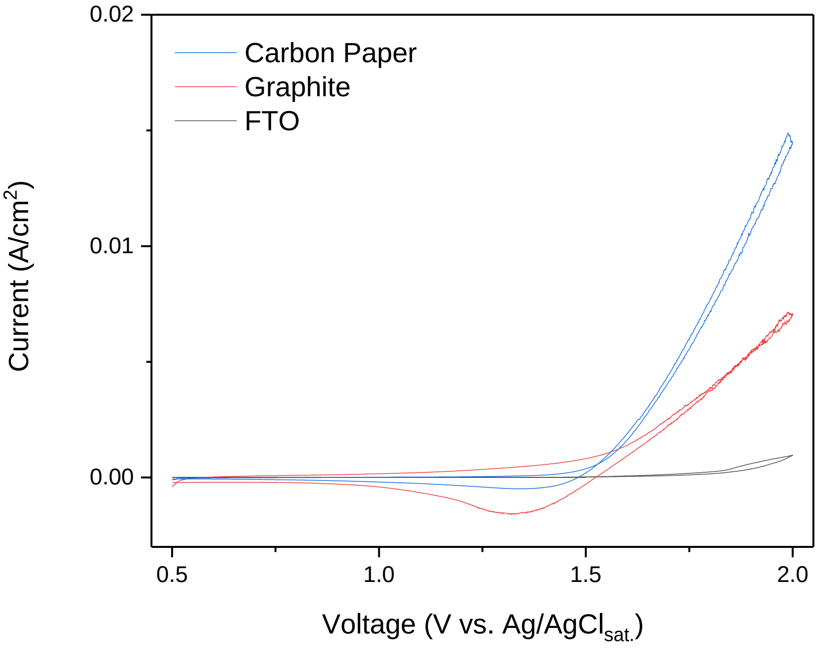

Figure S9. Cyclic voltammetry (CV) traces for different electrode materials (3-electrode setup, working electrode: carbon paper, graphite rod or FTO-coated glass sheet, reference electrode: Ag/AgCl$_{sat}$, counter electrode: Pt foil (2 cm^2), 24 mL of 10 mg mL^{-1} succinic acid solution in 0.1 M HNO_3, set to pH 10, scan rate 25 mV s^{-1}, 25 °C, under stirring).

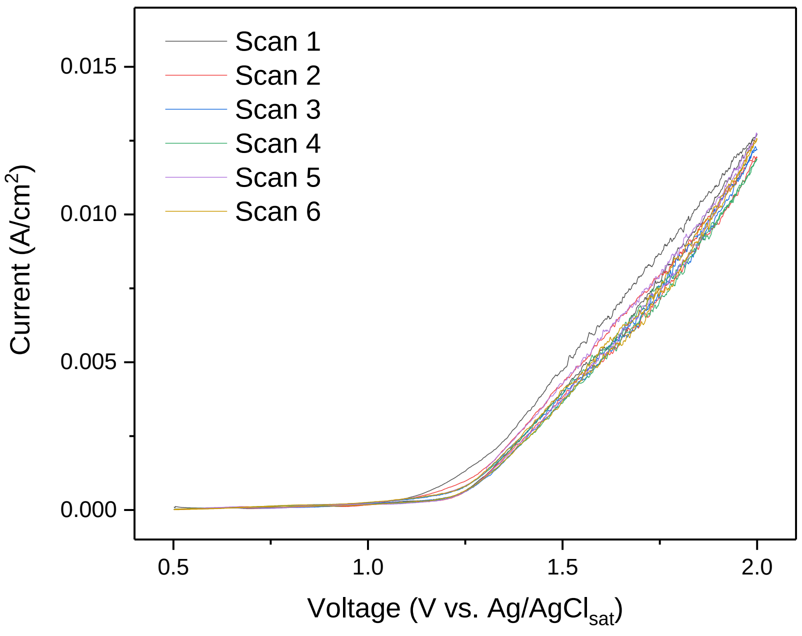

Figure S10. Cyclic voltammetry (CV) traces for carbon paper electrode, 3-electrode setup, reference electrode: Ag/AgCl$_{sat}$, counter electrode: Pt foil (2 cm^2), 24 mL of 10 mg mL^{-1} succinic acid solution in 0.1 M HNO_3, set to pH 10, scan rate 25 mV s^{-1}, 25 °C, under stirring.

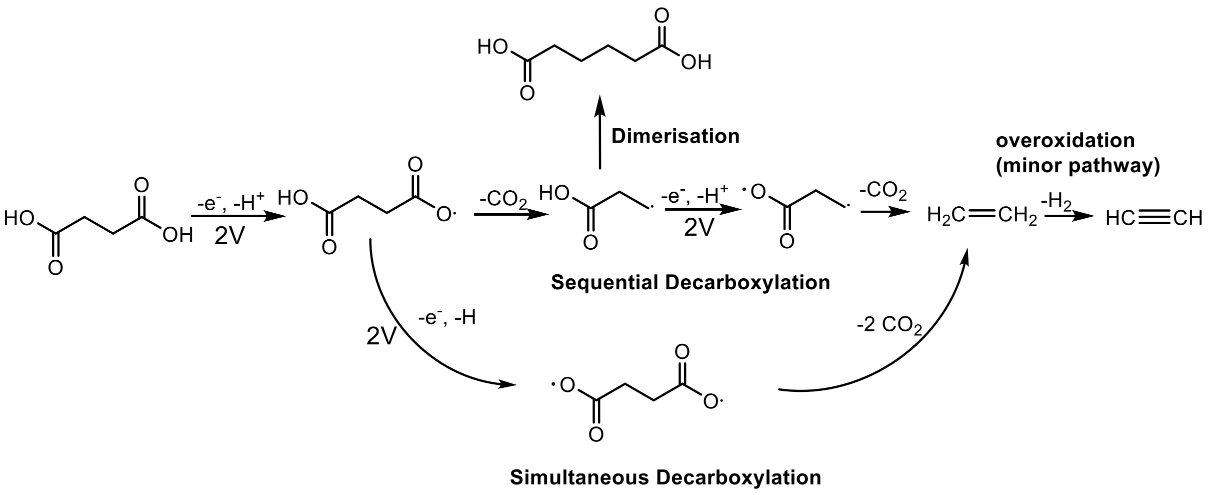

Figure S11. Reaction mechanism of electrocatalytic decarboxylation of succinic acid. The mechanism for this reaction does most likely not follow a classical Kolbe reaction or a Hofer-Moest pathway, which would proceed via formation of a carbocation, although the latter mechanism would be possible in theory.[8] The proposed mechanism suggests either a consecutive or a simultaneous oxidation of the two carboxylic acid groups forming intermediate radicals near the anode.[9]

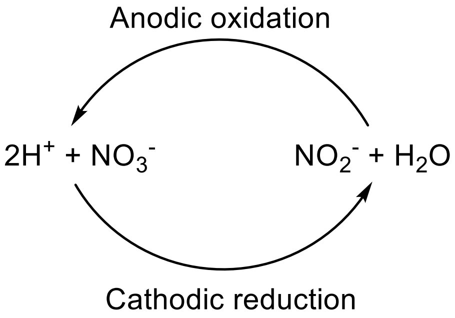

Figure S12. Nitrate/nitrite cycle.

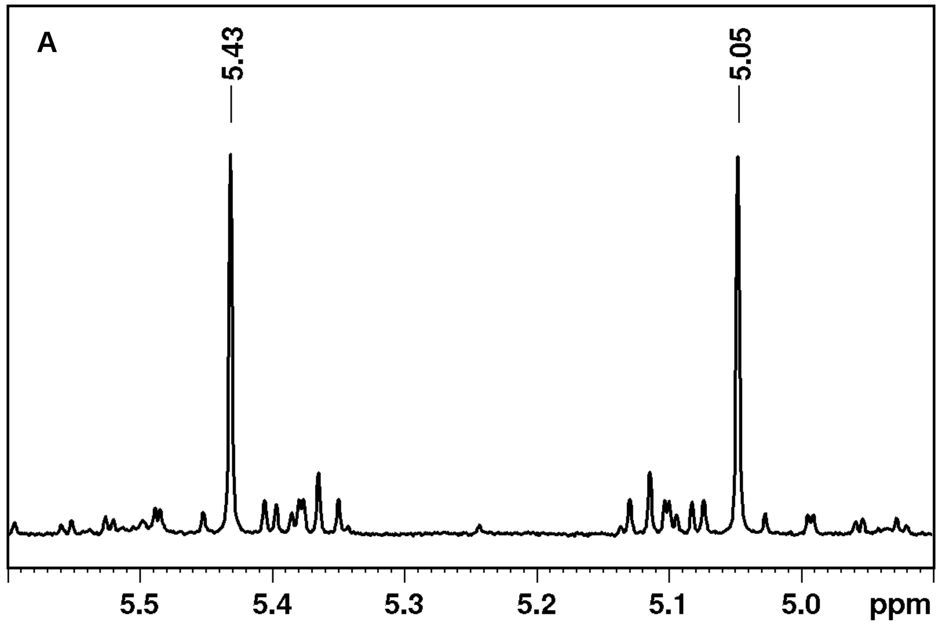

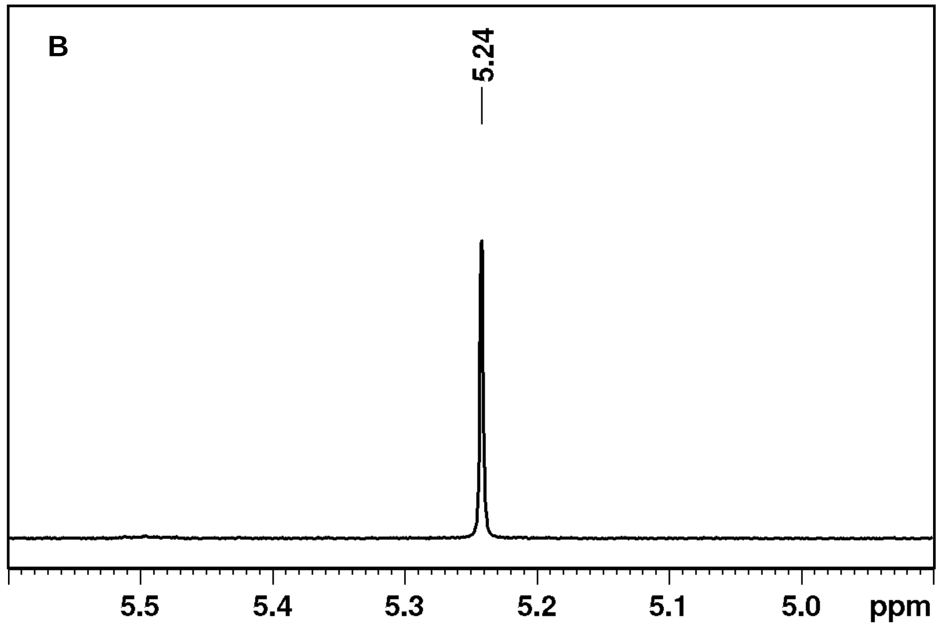

Figure S13. ^{1}H-NMR spectra of ethylene generated by electrocatalytic tests. (A) ^{13}C labelled succinic acid with a carbon paper working electrode, (B) unlabeled succinic acid with a carbon paper working electrode (3-electrode set up, working electrode: Carbon paper (2 cm^2 electrode area), counter electrode: Pt foil (2 cm^2 electrode area), reference electrode: Ag/AgCl$_{sat}$: 24 mL of 3.3 mg mL^{-1} succinic acid solution (normal or ^{13}C labelled) in 0.1 M HNO_3 set to pH 10 with 10 M NaOH. The ^{13}C labelled succinic acid yields ^{13}C labelled ethylene, causing a splitting of the proton-signal at 5.2 ppm (observed with the unlabeled succinic acid in (B)) into two signals at 5.4 and 5.0 ppm (A), coupling constant J=154 Hz.

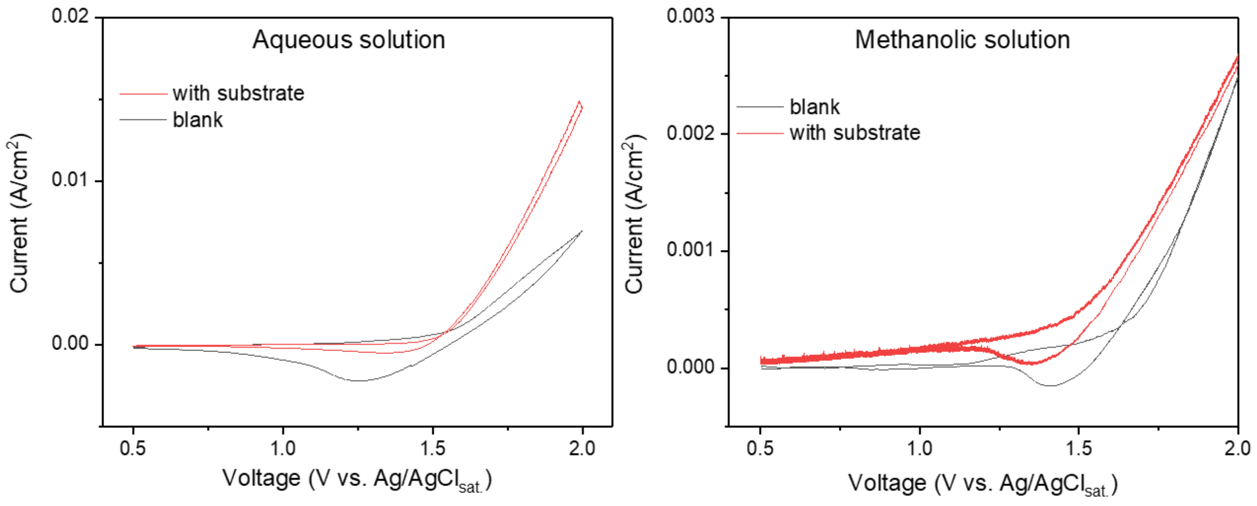

Figure S14. Blank cyclic voltammetry (CV) traces employing a 3-electrode setup. Working electrode: carbon paper, reference electrode: Ag/AgCl$_{sat}$, counter electrode: Pt foil (2 cm^2), 16 mL methanol and 6 mL mL 0.1 M HNO_3 set to pH 10 or 24 mL 0.1 M HNO_3 set to pH 10, containing 10 mg mL^{-1} succinic acid for "substrate solution", scan rate 25 mV sec^{-1}, 25 °C, under stirring.

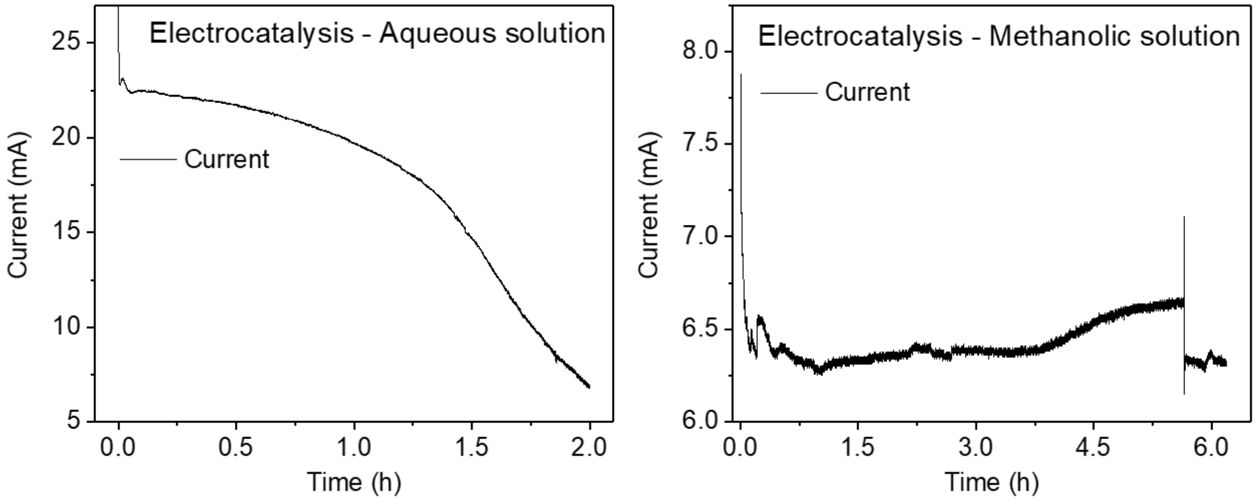

Figure S15. Chronoamperometric tests, two-electrode set up, working electrode: carbon paper (2 cm^2 electrode area), counter electrode: Pt foil (2 cm^2 electrode area), For aqueous conditions: 8 mL PE decomposition solution and 16 mL H_2O was set to pH 10 with 10 M NaOH; for methanolic conditions: 8 mL of PE decomposition solution set to pH 4 is diluted with 16 mL methanol, applied voltage 5 V, reaction time: until ca. 130±25 C (= 1±0.2 Faradaic equivalent) has passed. In aqueous condition, deactivation of the electrode starts at around 1h, while in the methanolic solution the current stays comparably constant. Table S15 shows that in aqueous solution oxidative degradation of the carbon electrode is comparably high, which is a likely mechanism for electrode deactivation. This oxidative degradation is significantly reduced in methanolic solutions, due to the lower current density.

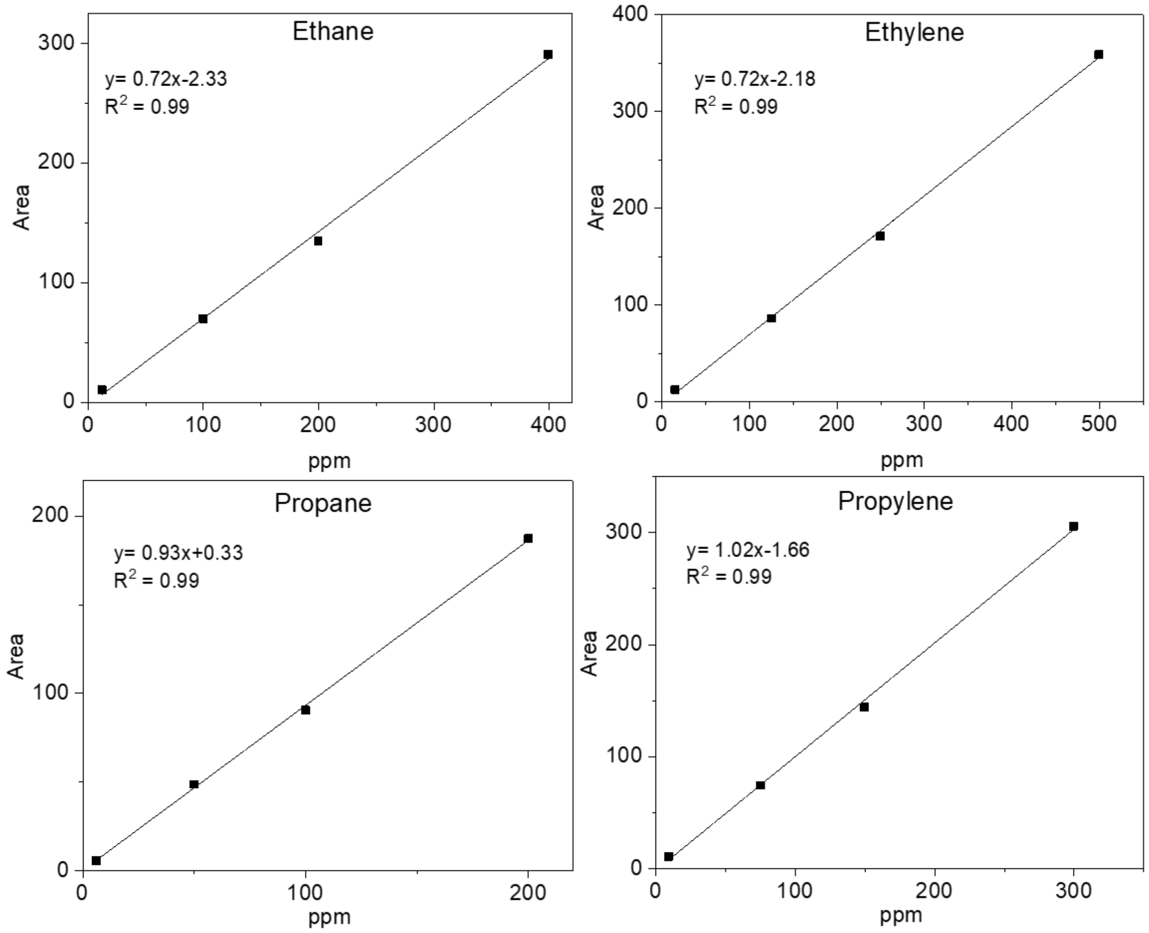

Figure S16. Calibration curves for GC measurements

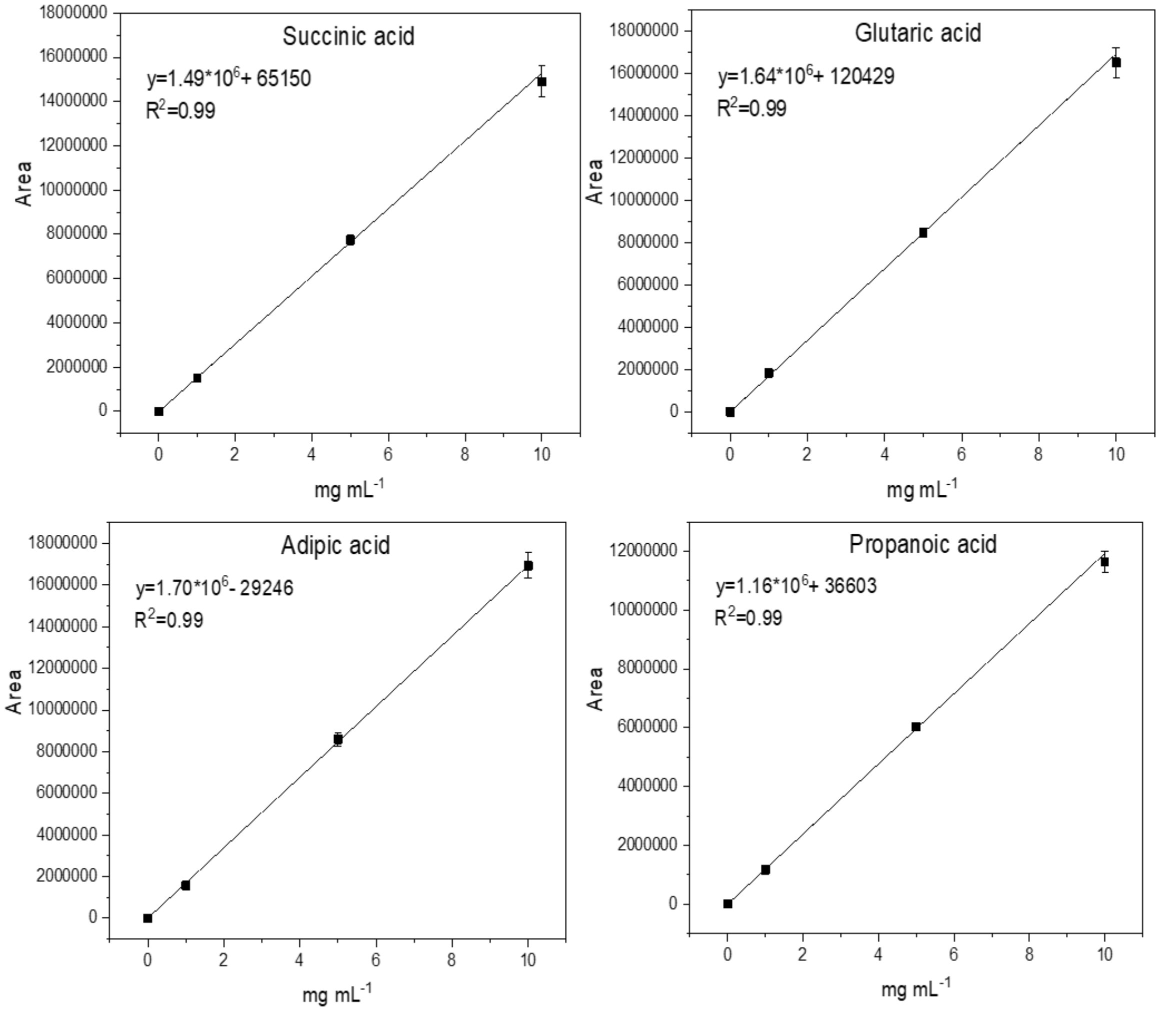

Figure S17. Calibration curves for HPLC measurements

Table S1. Yield of 300 mg polyethylene thermally treated with 11 mL 6% aqueous HNO_3.

	Yield (mmol)					%
**Conditions**	**Acetic acid**	**Propanoic acid**	**Succinic acid**	**Glutaric acid**	**Adipic acid**	**Carbon yield**
180 °C, 4h	0.44 ±0.01	0.08±0.01	0.88±0.01	0.45±0.01	0.19±0.01	38±1
180 °C, 8h	0.47±0.01	0.07±0.01	0.92±0.02	0.48±0.01	0.19±0.01	39±1
160 °C, 8h	0.27±0.01	0.07±0.01	0.66±0.02	0.38±0.01	0.22±0.01	31±1
180 °C, 4h 12% HNO_3	0.31±0.04	0.05±0.01	0.81±0.12	0.43±0.05	0.17±0.04	34±2
180 °C, 4h +10mg/mL Cu	0.42±0.01	0.10±0.01	0.69±0.02	0.46±0.01	0.27±0.01	37±1
180 °C, 4h, different PE	0.42±0.02	0.12±0.01	0.82±0.02	0.42±0.02	0.15±0.01	35±1

Table S2. Nitrate quantification by ion chromatography. Samples were diluted 1:10000 with MilliQ water before ion chromatography. The stated nitrate concentration gives the concentration in the original solution (before dilution).

**Sample**	**Area (µS cm^{-1} min^{-1})**	**NO_3^- concentration (mol L^{-1})**
Standard 0.8 M (3% HNO_3)	0.386	0.8
Standard 1.6 M (Before PE decomp.= 6% HNO_3)	0.752	1.6
Reaction solution after PE decomposition	0.065	0.1
Reaction solution after electrochemical reaction	0.071	0.1

Table S3. Product yields of time resolved photocatalytic experiments using AM1.5G, 100 mW cm^{-2} irradiation at 25 °C, with 4 mg P25|Pt or $^{NCN}CN_x$|Pt, 2 mL of 10 mg mL^{-1} succinic acid solution in 0.1 M HNO_3 set to different pH values with 10 M NaOH, no carbon mass balance was determined, as the generated CO_2 was absorbed in the solution at pH values > 4, n.d. = not detected.

	Reaction rate [µmol g_{cat}^{-1} h^{-1}] (mean (N=3))				
**Catalyst + pH**	**Ethylene**	**Ethane**	**Propanoic acid**	**Adipic acid**	**Hydrogen**
P25|Pt (pH 1)	n.d.	15.5±0.3	495.8±26.7	8.3±1.3	82.3±21.7
P25|Pt (pH 4)	n.d.	56.3±15.0	964.7±158.3	23.5±2.2	242.1±62.9
P25|Pt (pH 7)	n.d.	n.d.	27.1±1.4	n.d.	120.1±8.9
P25|Pt (pH 10)	n.d.	n.d.	14.3±8.8	3.3±0.7	72.9±18.4
$^{NCN}CN_x$|Pt (pH 1)	0.2±0.03	0.2±0.09	n.d.	n.d.	55.9±17.6
$^{NCN}CN_x$|Pt (pH 4)	1.3±0.1	7.2±0.9	176.7±10.0	25.2±0.2	137.1±8.3
$^{NCN}CN_x$|Pt (pH 7)	0.15±0.06	0.13±0.06	n.d.	n.d.	6.5±1.7
$^{NCN}CN_x$|Pt (pH 10)	n.d.	n.d.	n.d.	n.d.	n.d.

Table S4. Product yields of photocatalytic experiments using AM 1.5G, 100 mW cm^{-2} irradiation at 25 °C, with 4 mg catalyst, 2 mL of 10 mg mL^{-1} succinic acid solution in 0.1 M HNO_3 set to pH 4, irradiation time 24 h, n.d. = not detected.

	**Reaction rate [μmol g_{cat}^{-1} h^{-1}] (mean (N=3))**						
**Catalyst (atmosphere)**	**Ethylene**	**Ethane**	**Propanoic acid**	**Adipic acid**	**Hydrogen**	**CO_2**	**Carbon mass balance**
P25|Pt (H_2)	n.d.	40.3±17.3	995.7±81.2	15.2±10.7	-	832.0±98.4	98%
$^{NCN}CN_x$|Pt (H_2)	1.2±0.1	9.7±0.3	196.8±19.3	23.8±3.2	-	230.8±54.7	93%
P25|Pt (N_2)	n.d.	56.3±15.0	964.7±158.3	23.5±2.2	242.1±62.9	965.0±34.2	93%
$^{NCN}CN_x$|Pt (N_2)	1.3±0.1	7.2±0.9	176.7±10.0	25.2±0.2	137.1±8.3	189.2±11.6	94%
P25 (N_2)	0.5±0.2	1.6±0.4	347.7±40.7	19.2±1.1	1.5±0.9	267.1±28.1	92%
$^{NCN}CN_x$ (N_2)	0.3±0.1	0.3±0.2	11.8±3.1	n.d.	1.2±0.4	28.2±4.1	100%
P25|MoS(N_2)	0.07±0.06	1.3±0.1	128.6±48.4	n.d.	n.d.	141.2±0.7	95%
$^{NCN}CN_x$|MoS(N_2)	1.0±0.3	3.5±2.3	95.0±38.6	n.d.	n.d.	102.8±36.9	103%
P25|Pt (N_2) + $[CoCl(NH_3)_5]Cl_2$	0.51±0.06	n.d.	8.3	n.d.	n.d.	70.2±19.5	100%
$^{NCN}CN_x$|Pt (N_2) + $[CoCl(NH_3)_5]Cl_2$	7.1±0.7	1.5±0.4	64.8	6.1	n.d.	175.4±10.6	102%
P25|Pt (N_2), propanoic acid	n.d.	338.0±40.2	n.d.	n.d.	76.7±33.7	n.d.	n.d.
$^{NCN}CN_x$|Pt (N_2), propanoic acid	5.3±0.9	219.5±30.0	n.d.	n.d.	129.9±9.3	n.d.	n.d.
P25|Pt (N_2) no light	n.d.	n.d.	n.d.	n.d.	n.d.	n.d.	n.d.
$^{NCN}CN_x$|Pt (N_2) no light	n.d.	n.d.	n.d.	n.d.	n.d.	n.d.	n.d.
P25|Pt (N_2) no succinic acid	n.d.	n.d.	n.d.	n.d.	n.d.	n.d.	n.d.
$^{NCN}CN_x$|Pt (N_2) no succinic acid	n.d.	n.d.	n.d.	n.d.	n.d.	n.d.	n.d.
No catalyst	n.d.	n.d.	n.d.	n.d.	n.d.	n.d.	n.d.

Table S5. Product yields of time resolved photocatalytic experiments using AM 1.5G, 100 mW cm^{-2} irradiation at 25 °C, with 4 mg P25|Pt or $^{NCN}CN_x$|Pt, 2 mL of 10 mg mL^{-1} succinic acid solution in 0.1 M HNO_3 set to pH 4, n.d. = not detected.

	Amount of product formed [mmol g_{cat}^{-1}] (mean (N=3))						
**Catalyst + time**	**Ethylene**	**Ethane**	**Propanoic acid**	**Adipic acid**	**Hydrogen**	**CO_2**	**Carbon mass balance**
P25|Pt (8 h)	n.d.	0.13±0.02	8.8±0.5	0.4±0.2	1.1±0.2	6.2±0.3	95%
P25|Pt (24 h)	n.d.	1.4±0.4	23.1±3.8	0.6±0.05	5.8±1.5	23.1±0.8	93%
P25|Pt (48 h)	n.d.	4.2±1.6	29.3±3.1	0.4±0.05	11.9±2.8	34.2±1.5	92%
P25|Pt (72 h)	n.d.	10.0±2.6	20.8±1.7	n.d.	20.0±0.9	46.5±5.6	78%
$^{NCN}CN_x$|Pt (8 h)	0.007±0.001	0.04±0.003	1.3±0.2	0.2±0.04	1.2±0.1	2.1±0.1	98%
$^{NCN}CN_x$|Pt (24 h)	0.05±0.001	0.29±0.006	5.2±1.2	0.5±0.06	4.0±0.4	6.0±0.7	90%
$^{NCN}CN_x$|Pt (48 h)	0.1±0.01	1.01±0.08	8.1±0.7	0.8±0.02	7.8±0.7	11.2±1.2	88%
$^{NCN}CN_x$|Pt (72 h)	0.15±0.02	1.9±0.4	8.6±1.0	0.7±0.01	10.4±1.9	15.9±1.3	82%

Table S6. External quantum yield (EQY) measurements from photoreforming of polymers. Conditions: ultra-sonicated P25|Pt or $^{NCN}CN_x$|Pt (3 mg), 1.5 mL succinic acid solution (10 mg mL^{-1}), all in a sealed quartz cuvette (path length 1 cm, internal volume 3.83 mL) under anaerobic conditions. Samples were irradiated with monochromatic light (λ = 360 nm for P25|Pt or 400 nm for $^{NCN}CN_x$|Pt, full-width at half maximum: 5, intensity taken as the average of the intensities measured at the beginning and end of the experiments) over an area of 0.28 cm^2 for 24 h, before samples were taken for GC analysis, n.d. = not detected.

**Catalyst**	**Light intensity (mW cm^{-2})**	**Ethylene (µmol)**	**Ethane (µmol)**	**EQY Ethylene (%)**	**EQY Ethane (%)**
P25|Pt	10.8±3.1	n.d.	0.17±0.09	n.d.	0.42±0.09
$^{NCN}CN_x$|Pt	12.2±2.0	0.025±0.001	0.019±0.005	0.053±0.001	0.04±0.002

Table S7. Product yields of photocatalytic experiments using AM1.5G, 100 mW cm^{-2} irradiation at 25 °C, with 4 mg catalyst, 2 mL of 11 mg mL^{-1} glutaric acid solution in 0.1 M HNO_3 set to pH 4, irradiation time 24 h.

	**Reaction rate [µmol g_{cat}^{-1} h^{-1}] (mean (N=3))**					
**Catalyst**	**Propylene**	**Propane**	**Butyric acid**	**Hydrogen**	**CO_2**	**Carbon mass balance**
P25|Pt	0.04±0.04	17.1±5.1	491.1±67.8	150.6±30.8	371.4±46.5	80%
$^{NCN}CN_x$|Pt	0.14±0.06	4.9±1.2	111.1±41.6	113.6±16.4	166.3±20.1	84%

Table S8. Product yields of photocatalytic experiments using AM 1.5G, 100 mW cm^{-2} irradiation at 25 °C, with 4 mg P25|Pt and 0.2 mL PE decomposition solution and 1.8 mL H_2O, set to pH 4, reaction time 96 h.

	**Amount of product formed [mmol g_{cat}^{-1}] (mean (N=3))**					
**Catalyst + time**	**Ethylene**	**Ethane**	**Propylene**	**Propane**	**Hydrogen**	**CO_2**
P25|Pt (96 h)	0.017	0.25	0.007	0.14	6.3	5.9

**Overall yield gaseous hydrocarbons from PE**	**1.0%**
**Overall yield of CO_2 from PE**	**6.1%**

Calculation of hydrocarbon or CO_2 yield:

$$Y_{total}(\%) = 100 * \frac{mol_{gas}}{V_{PE}C_{PE}}$$

Y_{total}…Total hydrocarbon (ethane+ ethylene + propane + propylene) or CO_2 yield (in %) from PE

mol_{gas}…Evolved amount of gas (mol carbon of ethane + ethylene + propane+ propylene or CO_2) during photocatalysis

V_{PE}…Volume (mL) of pure converted polyethylene decomposition solution

C_{PE}…Starting concentration of PE (mol carbon mL^{-1}) for the polyethylene decomposition solution

Table S9. Product yields in photocatalytic experiments using custom made flow system, with an irradiated area of 25 cm^2, 100 mW cm^{-2}, AM 1.5G irradiation from the back at 25 °C, with P25|Pt or $^{NCN}CN_x$|Pt deposited on glass sheets, 50 mL of PE decomposition solution set to pH 4, As the reaction time is circulated through the reactor and the reservoir, the actual irradiation time is 0.6 the actual experiment duration. Stated here are the actual irradiation times.

	**Amount of product formed (µmol m^{-2}_{cat})**			
**Catalyst (time)**	**Ethylene**	**Ethane**	**Propylene**	**Propane**
P25|Pt (0.6 d)	n.d.	25.5±2.7	n.d.	15.0±5.7
P25|Pt (1.2 d)	n.d.	36.7±9.1	n.d.	31.9±11.7
P25|Pt (1.8 d)	n.d.	55.8±8.9	n.d.	38.5±13.8
$^{NCN}CN_x$|Pt (0.6 d)	13.4±0.7	35.8±1.3	13.2±5.7	19.1±6.7
$^{NCN}CN_x$|Pt (1.2 d)	45.4±3.8	53.2±6.4	17.6±5.8	30.2±3.9
$^{NCN}CN_x$|Pt (1.8 d)	69.2±5.8	77.9±7.1	19.1±5.5	40.7±7.3

Table S10. Chronoamperometric tests with varying electrode material and potential, 3 electrode setup, Working electrode: Carbon Paper, Graphite rod or FTO glass sheet, Reference electrode: Ag/AgCl$_{sat}$, Counter electrode: Pt foil (2 cm^2), 24 mL of 10 mg/mL succinic acid solution in 0.1 M HNO_3, set to pH 4, Reaction time: 20 min, Potential given vs. Ag/AgCl$_{sat}$,n.d. = not detected.

**Conditions**	**Ethylene**		**Acetylene**		**Adipic acid**		**Hydrogen**	
	µmol cm^{-2} h^{-1}	Faradaic yield (%)	µmol cm^{-2} h^{-1}	Faradaic yield (%)	µmol cm^{-2} h^{-1}	Faradaic yield (%)	µmol cm^{-2} h^{-1}	Faradaic yield (%)
Carbon Paper 1.5V	0.3±0.1	10.1±1.1	n.d.	n.d.	n.d.	n.d.	1.6±0.6	53.8±13.4
Carbon Paper 2.0V	28.5±6.9	12.7±1.7	0.9±0.3	0.4±0.2	24.5±2.9	10.9±0.3	105.4±24.9	47.0±6.4
Carbon Paper 2.5V	48.1±7.2	9.4±0.8	0.9±0.8	3.8±3.5	36.8±5.7	7.6±1.1	311.8±89.9	71.1±9.5
Carbon Paper 3.0V	35.0±9.5	5.5±1.3	7.5±0.8	1.2±0.1	28.1±8.7	5.1±2.1	306.4±117.0	49.6±21.9
Graphite 1.5V	0.5±0.3	10.8±0.5	n.d.	n.d.	n.d.	n.d.	2.1±1.3	51.1±9.9
Graphite 2.0V	6.9±1.2	5.6±1.0	0.4±0.3	0.3±0.2	n.d.	n.d.	84.1±9.6	68.3±9.9
Graphite 2.5V	14.6±6.1	4.1±2.1	2.5±1.1	0.8±0.3	10.0±5.7	2.0±2.0	204.6±11.2	62.8±3.5
Graphite 3.0V	6.4±3.0	0.8±0.6	2.3±1.1	0.3±0.1	10.3±3.5	1.8±0.7	382.5±98.1	57.7±16.8
FTO 1.5V	n.d.	n.d.	n.d.	n.d.	n.d.	n.d.	n.d.	n.d.
FTO 2.0V	0.3±0.2	6.6±3.5	n.d.	n.d.	n.d.	n.d.	4.1±2.5	70.9±14.3
FTO 2.5V	1.3±0.9	2.4±0.4	n.d.	n.d.	n.d.	n.d.	30.0±26.1	56.3±18.0
FTO 3.0V	1.1±0.7	0.8±0.6	n.d.	n.d.	n.d.	n.d.	81.1±4.1	57.7±16.8

Table S11. Chronoamperometric tests, with varying electrode material and pH value. Three-electrode setup, working electrode: carbon Paper, graphite rod or FTO-coated glass sheet, reference electrode: Ag/AgCl$_{sat.}$, counter electrode: Pt foil (2 cm^2), 24 mL of 10 mg mL^{-1} succinic acid solution in 0.1 M HNO_3, set to different pH values with 10 M NaOH, reaction time: 20 min, applied potential 2 V vs. Ag/AgCl$_{sat.}$,n.d. = not detected

**Conditions**	**Ethylene**		**Acetylene**		**Adipic acid**		**Hydrogen**	
	µmol $cm^{-2}{}_{cat}$ h^{-1}	Faradaic yield (%)	µmol $cm^{-2}{}_{cat}$ h^{-1}	Faradaic yield (%)	µmol $cm^{-2}{}_{cat}$ h^{-1}	Faradaic yield (%)	µmol $cm^{-2}{}_{cat}$ h^{-1}	Faradaic yield (%)
Carbon Paper pH 1	5.3±1.3	3.0±1.1	0.7±0.3	0.4±0.1	8.1±0.9	2.7±2.3	65.1±45.8	39.9±33.5
Carbon Paper pH 4	28.5±6.9	12.7±1.7	0.9±0.3	0.4±0.2	24.5±2.9	10.9±0.3	105.4±24.9	47.0±6.4
Carbon Paper pH 7	88.1±5.6	27.7±8.6	0.5±0.4	0.1±0.1	18.2±9.6	5.1±1.0	256.7±49.7	78.0±13.8
Carbon Paper pH 10	84.1±34.3	28.6±1.9	1.5±0.5	0.4±0.2	18.9±9.6	6.3±5.4	242.3±90.1	83.4±6.3
Graphite pH 1	6.1±1.0	2.0±0.5	1.6±0.2	0.6±0.2	n.d.	n.d.	145.7±82.5	47.4±23.9
Graphite pH 4	6.9±1.2	5.6±1.0	0.4±0.3	0.3±0.2	n.d.	n.d.	84.1±9.6	68.3±9.9
Graphite pH 7	27.9±19.9	9.0±6.5	1.6±0.2	0.3±0.2	17.9±8.2	7.4±5.4	140.8±29.2	45.4±8.3
Graphite pH 10	62.6±24.8	18.4±6.5	3.8±2.2	1.1±0.4	19.1±1.7	6.2±2.2	221.5±36.6	64.7±8.3
Carbon Paper pH 4 no succinic acid	n.d.	n.d.	n.d.	n.d.	n.d.	n.d.	372.1±37.7	93.7±5.8

Table S12. Detection of oxygen with fluorometric sensor, two-electrode set up, Working electrode: carbon paper (2 cm^2 electrode area), single compartment cell, 24 mL reaction solution.

**Reaction conditions**	**O_2 Faradaic yield (%)**
0.05 M NaOH	86
0.1 M HNO_3 set to pH 10 with 10 M NaOH	< 2
3.3 mg succinic/mL in 0.1 M HNO_3, pH10	5

Table S13. Chronopotentiometric test using three-electrode setup. Working electrode: carbon Paper (2 cm^2), reference electrode: Ag/AgCl$_{sat.}$, counter electrode: Pt foil (2 cm^2), 24 mL of 11 mg mL^{-1} glutaric acid solution in 0.1 M HNO_3, set to pH 4, Reaction time: 20 min, Set current: 30 mA.

**Conditions**	**Propylene**		**Hydrogen**	
	µmol cm^{-2} h^{-1}	Faradaic yield (%)	µmol cm^{-2} h^{-1}	Faradaic yield (%)
Carbon Paper, pH4	10.6	3.8	214.8	76.8

Table S14. Optimized conditions for chronoamperometric tests, two-electrode set up, working electrode: carbon paper (2 cm^2 electrode area), counter electrode: Pt foil (2 cm^2 electrode area), single compartment cell, For aqueous conditions: 24 mL of 3.3 mg mL^{-1} succinic acid solution in 0.1 M HNO_3 set to pH 10 with 10 M NaOH; For methanolic conditions: 8 mL of 10 mg mL^{-1} succinic acid solution in 0.1 M HNO_3 set to pH 4 is diluted with 16 mL methanol, applied voltage 5 V, reaction time: Until ca. 130 C (= 1 Faradaic equivalent) was reached

Conditions (N=3)		**pH 10**	**2:1 Methanol**
**Ethylene**	µmol cm^{-2}_{cat} h^{-1}	47.9±4.3	21.0±5.2
	Faradaic yield (%)	27.1±4.7	38.3±8.7
**Acetylene**	µmol cm^{-2}_{cat} h^{-1}	4.3±3.4	0.2±0.1
	Faradaic yield (%)	3.1±0.9	0.3±0.04
**Adipic acid**	µmol cm^{-2}_{cat} h^{-1}	5.8±0.2	5.4±0.9
	Faradaic yield (%)	3.2±0.6	9.6±1.7
**Hydrogen**	µmol cm^{-2}_{cat} h^{-1}	161.9±31.4	40.4±5.2
	Faradaic yield (%)	89.6±6.4	73.6±7.8

Table S15. Consumption of auxiliary reactants. To determine the overoxidation of methanol and the carbon electrode to CO_2, chronoamperometric tests were conducted, two-electrode set up, working electrode: carbon paper (2 cm^2 electrode area), counter electrode: Pt foil (2 cm^2 electrode area), single compartment cell, 24 mL 0.1 M HNO_3 set to pH 10 (for carbon electrode decomposition only) or 8 mL (0.1 M HNO_3 set to pH 10) and 16 mL MeOH (for combined carbon electrode and MeOH oxidation), applied voltage 5 V, reaction time: until ca. 60 C have passed. The reaction atmosphere was manually sampled, and the evolved CO_2 was quantified by GC. Faradaic yield for the overoxidation of MeOH or the carbon electrode to CO_2 was calculated.

**Compound**	**Findings**	**Consumption**
HNO_3	> 90% consumed during thermal treatment (see table S2), negligible consumption during electrocatalysis.	0.055 mol HNO_3 g^{-1} of decomposed polyethylene
Methanol	Slowly oxidized during electrocatalysis with a maximum contribution to the Faradaic yield of 4%.	Max. 0.1 mol MeOH mol^{-1} consumed in parasitic solvent oxidation
Carbon electrode	Moderately oxidized during electrocatalysis in aqueous solution, with a maximum contribution of 23% Faradaic yield.<br><br>Slowly oxidized during electrocatalysis in methanolic solution with a maximum contribution to the Faradaic yield of 4%.	2-10 g carbon electrode mol^{-1} formed gaseous product

As the overoxidation of the carbon electrode and the methanol occurs simultaneously, their respective contributions cannot be separated. The given value for methanol overoxidation also includes the carbon electrode oxidation and hence the calculated Faradaic yield of 4% for the methanol overoxidation is the maximal value. The overoxidation of carbon electrodes alone, was determined in aqueous reaction solutions, where it reaches 23% of the faradaic yield (which is most likely the cause for catalyst deactivation under these conditions). In methanolic solution the electrode overoxidation must be significantly lower (as the combined methanol and electrode overoxidation reached a max. faradaic yield of 4%).

Table S16. Optimized conditions for chronoamperometric tests, two-electrode set up, working electrode: carbon paper (2 cm^2 electrode area), counter electrode: Pt foil (2 cm^2 electrode area), For aqueous conditions: 8 mL PE decomposition solution and 16 mL H_2O was set to pH 10 with 10 M NaOH; for methanolic conditions: 8 mL of PE decomposition solution set to pH 4 is diluted with 16 mL methanol, applied voltage 5 V, reaction time: until ca. 130±25 C (= 1±0.2 Faradaic equivalent) has passed, n.d.= not detected.

Conditions (N=3)		**pH 10**	**2:1 Methanol**
**Ethylene**	µmol cm^{-2}_{cat} h^{-1}	6.0±0.9	5.0±0.4
	Faradaic yield (%)	3.8±0.5	9.0±0.7
**Acetylene**	µmol cm^{-2}_{cat} h^{-1}	1.9±0.3	n.d.
	Faradaic yield (%)	1.2±0.2	n.d.
**Propylene**	µmol cm^{-2}_{cat} h^{-1}	1.1±0.2	2.2±0.1
	Faradaic yield (%)	0.7±0.1	3.9±0.3
**Butylene**	µmol cm^{-2}_{cat} h^{-1}	<0.3	<0.3
	Faradaic yield (%)	< 0.2%	< 0.2%
**Hydrogen**	µmol cm^{-2}_{cat} h^{-1}	159.0±9.8	36.7±3.9
	Faradaic yield (%)	100±1	65.3±7.1

Table S17. Optimized conditions for chronoamperometric tests at higher conversions, two-electrode set up, working electrode: carbon paper (2 cm^2 electrode area), counter electrode: Pt foil (2 cm^2 electrode area), 1 mL of PE decomposition solution and 7 mL H_2O set to pH 10 is diluted with 16 mL methanol, applied voltage 5 V, reaction time: until ca. 150±25 C (= 1±0.2 Faradaic equivalent) has passed.

Conditions (N=3)		**Reaction rate**
**Ethylene**	µmol cm^{-2}_{cat} h^{-1}	4.2±0.7
	Faradaic yield (%)	13.6±3.4
**Propylene**	µmol cm^{-2}_{cat} h^{-1}	0.7±0.1
	Faradaic yield (%)	3.2±1.0

**Overall yield gaseous hydrocarbons from PE**	**7.6%**
**Yield CO_2 decarboxylation side product from PE**	**13.5%**

Calculation of overall yield:

$$Y_{total}(\%) = 100 * \frac{mol_{gas}}{V_{PE}C_{PE}}$$

Y_{total}…Total hydrocarbon (ethylene + propylene) yield (in %) from PE

mol_{gas}…Evolved amount of gas (mol carbon of ethylene + propylene) during electrocatalysis

V_{PE}…Volume (mL) of pure converted polyethylene decomposition solution

C_{PE}…Starting concentration of PE (mol carbon mL^{-1}) for the polyethylene decomposition solution

Calculation of CO_2 yield:

$$Y_{CO2}(\%) = 100 * \frac{mol_{CO2}}{V_{PE}C_{PE}}$$

Y_{CO2}…Total CO_2 yield (in %) from PE

mol_{CO2}…Evolved amount of CO_2 (mol carbon of CO_2) during electrocatalysis.

V_{PE}…Volume (mL) of pure converted polyethylene decomposition solution

C_{PE}…Starting concentration of PE (mol carbon mL^{-1}) for the polyethylene decomposition solution

Table S18. Comparison of the reported process with other plastic waste conversion processes.

**Process**	**Reaction temperature**	**Byproducts**	**Complexity of product mixture**	**Plastic to Ethylene and Propylene yield**
Pyrolysis and steam reforming	450-600 °C (pyrolysis)[10]<br>600-800 °C (steam cracking)[11]	CO_2, CO, CH_4, C_2H_6, C_3H_8, C_4-C_5 hydrocarbons aromatics,[12,13]	high	15-35%[10,14,15]
Gasification + syngas conversion	800-1000 °C (gasification)[16]<br>200-400 °C (syngas conversion)[17]	CO_2, CO, CH_4, C_4 olefins [17]	middle	< 40%[16,17]
This work	180 °C (decomposition) and 25 °C (electrochemical step)	CO_2	Low-middle	7.6%

Supporting References

(1) Rutten, O. W. J. S.; Sandwijk, A. V. A. N.; Weert, G. V. A. N. The Electrochemical Reduction of Nitrate in Acidic Nitrate Solutions. *J. Appl. Electrochem.* **1999**, *29*, 87–92. https://doi.org/10.1023/A:1003412613806

(2) Stang, C.; Harnisch, F. The Dilemma of Supporting Electrolytes for Electroorganic Synthesis: A Case Study on Kolbe Electrolysis. *ChemSusChem* **2016**, *9*, 50–60. https://doi.org/10.1002/cssc.201501407.

(3) McEnaney, J. M.; Blair, S. J.; Nielander, A. C.; Schwalbe, J. A.; Koshy, D. M.; Cargnello, M.; Jaramillo, T. F. Electrolyte Engineering for Efficient Electrochemical Nitrate Reduction to Ammonia on a Titanium Electrode. *ACS Sustain. Chem. Eng.* **2020**, *8*, 2672–2681. https://doi.org/10.1021/acssuschemeng.9b05983.

(4) Li, M.; Feng, C.; Zhang, Z.; Yang, S.; Sugiura, N. Treatment of Nitrate Contaminated Water Using an Electrochemical Method. *Bioresour. Technol.* **2010**, *101*, 6553–6557. https://doi.org/10.1016/j.biortech.2010.03.076.

(5) Partenheimer, W. Valuable Oxygenates by Aerobic Oxidation of Polymers Using Metal/Bromide Homogeneous Catalysts. *Catal. Today* **2003**, *81*, 117–135. https://doi.org/10.1016/S0920-5861(03)00124-X.

(6) Karlsson, S.; Hakkarainen, M.; Albertsson, A. C. Dicarboxylic Acids and Ketoacids Formed in Degradable Polyethylenes by Zip Depolymerization through a Cyclic Transition State. *Macromolecules* **1997**, *30*, 7721–7728. https://doi.org/10.1021/ma961489c.

(7) Uekert, T.; Bajada, M. A.; Schubert, T.; Pichler, C. M.; Reisner, E. Scalable Photocatalyst Panels for Photoreforming of Plastic, Biomass and Mixed Waste in Flow. *ChemSusChem* **2020**. https://doi.org/10.1002/cssc.202002580.

(8) Holzhäuser, F. J.; Creusen, G.; Moos, G.; Dahmen, M.; König, A.; Artz, J.; Palkovits, S.; Palkovits, R. Electrochemical Cross-Coupling of Biogenic Di-Acids for Sustainable Fuel Production. *Green Chem.* **2019**, *21*, 2334–2344. https://doi.org/10.1039/c8gc03745k.

(9) Meyers, J.; Kurig, N.; Gohlke, C.; Valeske, M.; Panitz, S.; Holzhäuser, F. J.; Palkovits, R. Intramolecular Biradical Recombination of Dicarboxylic Acids to Unsaturated Compounds: A New Approach to an Old Kolbe Reaction. *ChemElectroChem* **2020**, *7*, 4873–4878. https://doi.org/10.1002/celc.202001256.

(10) Miandad, R.; Barakat, M. A.; Aburiazaiza, A. S.; Rehan, M.; Nizami, A. S. Catalytic Pyrolysis of Plastic Waste: A Review. *Process Saf. Environ. Prot.* **2016**, *102*, 822–838. https://doi.org/10.1016/j.psep.2016.06.022.

(11) Rahimi, N.; Karimzadeh, R. Applied Catalysis A : General Catalytic Cracking of Hydrocarbons over Modified ZSM-5 Zeolites to Produce Light Olefins : A Review. *Applied Catal. A, Gen.* **2011**, *398*, 1–17. https://doi.org/10.1016/j.apcata.2011.03.009.

(12) Karaba, A.; Dvořáková, V.; Patera, J.; Zámostný, P. Journal of Analytical and Applied Pyrolysis Improving the Steam-Cracking Efficiency of Naphtha Feedstocks by Mixed / Separate Processing. *J. Anal. Appl. Pyrolysis* **2020**, *146*, 104768. https://doi.org/10.1016/j.jaap.2019.104768.

(13) Pralhad, V.; Chen, Y.; Neal, L.; Li, F. Intensification of Ethylene Production from Naphtha via a Redox Oxy-Cracking Scheme : Process Simulations and Analysis. *Engineering* **2018**, *4*, 714–721. https://doi.org/10.1016/j.eng.2018.08.001.

(14) Yang, M.; You, F. Comparative Techno-Economic and Environmental Analysis of Ethylene and Propylene Manufacturing from Wet Shale Gas and Naphtha. *Ind. Eng.*

Chem. Res. **2017**, *56*, 4038–4051. https://doi.org/10.1021/acs.iecr.7b00354.

(15) Sadrameli, S. M. Thermal / Catalytic Cracking of Liquid Hydrocarbons for the Production of Olefins : A State-of-the-Art Review II : Catalytic Cracking Review. *Fuel* **2016**, *173*, 285–297. https://doi.org/10.1016/j.fuel.2016.01.047.

(16) Lopez, G.; Artetxe, M.; Amutio, M.; Alvarez, J.; Bilbao, J.; Olazar, M. Recent Advances in the Gasification of Waste Plastics. A Critical Overview. *Renew. Sustain. Energy Rev.* **2018**, *82*, 576–596. https://doi.org/10.1016/j.rser.2017.09.032.

(17) Galvis, H. M. T.; Jong, K. P. De. Catalysts for Production of Lower Olefins from Synthesis Gas : A Review. *ACS Catal.* **2013**, *3*, 2130–2149. https://doi.org/10.1021/cs4003436

End of Supporting Information
